# Protective effect of compatible herbs in Jin-Gu-Lian formula against *Alangium chinense*-induced neurotoxicity *via* oxidative stress, neurotransmitter metabolisms, and pharmacokinetics

**DOI:** 10.3389/fphar.2023.1133982

**Published:** 2023-02-16

**Authors:** Dongyin Lian, Tengfei Chen, Lihua Yan, Hongping Hou, Shuangrong Gao, Qin Hu, Guangping Zhang, Han Li, Ling Song, Yunhang Gao, Yunxi Pu, Ying Chen, Bo Peng

**Affiliations:** ^1^ Institute of Chinese Materia Medica, China Academy of Chinese Medical Sciences, Beijing, China; ^2^ College of Life Sciences and Bio-Engineering, Beijing University of Technology, Beijing, China; ^3^ College of Letters and Science, University of California, Santa Barbara, CA, United States

**Keywords:** *Alangium chinense*, neurotoxicity, pharmacokinetics, cytochrome P450, oxidative stress, neurotransmitters

## Abstract

**Background:** *A. chinense* frequently used in Miao medicine to treat rheumatic diseases. However, as a famous toxic herb, *Alangium chinense* and its representative components exhibit ineluctable neurotoxicity, thus creating significant challenges for clinical application. The combined application with compatible herbs in Jin-Gu-Lian formula attenuates such neurotoxicity according to the compatible principle of traditional Chinese medicines.

**Purpose:** We aimed to investigate the detoxification of the compatible herbs in Jin-Gu-Lian formula on *A. chinense*-induced neurotoxicity and investigate its mechanism.

**Methods:** Neurobehavioral and pathohistological analysis were used to determine the neurotoxicity in rats administered with *A. chinense* extract (AC), extract of compatible herbs in Jin-Gu-Lian formula (CH) and combination of AC with CH for 14 days. The mechanism underlying the reduction of toxicity by combination with CH was assessed by enzyme-linked immunosorbent assays, spectrophotometric assays, liquid chromatography tandem-mass spectrometry and real-time reverse transcription-quantitative polymerase chain reaction.

**Results:** Compatible herbs attenuated the AC-induced neurotoxicity as evidenced by increased locomotor activity, enhanced grip strength, the decreased frequency of AC-induced morphological damage in neurons, as well as a reduction of neuron-specific enolase (NSE) and neurofilament light chain (NEFL) levels. The combination of AC and CH ameliorated AC-induced oxidative damage by modulating the activities of superoxide dismutase (SOD) and glutathione peroxidase (GSH-Px), and total antioxidant capacity (T-AOC). AC treatment significantly reduced the levels of monoamine and acetylcholine neurotransmitters in the brains of rats, including acetylcholine (Ach), dopamine (DA), 3,4-dihydroxyphenylacetic acid (DOPAC), homovanillic acid (HVA), norepinephrine (NE), and serotonin (5-HT). Combined AC and CH treatment regulated the abnormal concentrations and metabolisms of neurotransmitters. Pharmacokinetic studies showed that the co-administration of AC and CH significantly decreased plasma exposure levels of two main components of AC, as evidenced by the reduction of maximum plasma concentration (C_max_), area under the plasma concentration-time curve (AUC) compared to AC. In addition, the AC-induced downregulation in mRNA expression of cytochrome P450 enzymes was significantly reduced in response to combined AC and CH treatment.

**Conclusion:** Compatible herbs in Jin-Gu-Lian formula alleviated the neurotoxicity induced by *A. chinense* by ameliorating oxidative damage, preventing abnormality of neurotransmitters and modulating pharmacokinetics.

## 1 Introduction

*Alangium chinense* (Lour.) Harms (also known as Ba Jiao Feng) is derived from the *Alangium* genus of the Alangiaceae family and is widely distributed in the southwest of China (Hu et al., 2020). *Alangium chinense*, a routine and important Miao medicine in ethnomedicine, is used at a high frequency of more than 100 in 842 Miao medical formulas collected from relevant Miao medicine books, including Chinese Miao Medicine, Chinese Materia Medica of Miao Medicinal Volume, Medico-Miao and other Miao ethnic medicine books (Li et al., 2017). The properties and effects of *A. chinense* are described as dispelling pathogenic wind and eliminating dampness, relaxing tendon and freeing meridians, dispersing blood stasis and detumescence, as well as relieving pain. In traditional clinical treatment, *A. chinense* was used in combination with other herbal materials in many widely used folk prescriptions as a remedy for rheumatic diseases (Zheng et al., 2022), acroanesthesia and fractures, including Jin Gu Lian (JGL) capsules, Feng Shi Ding tablets and Xiao Bi Ling mixture (Meng et al., 2021). Previous experimental studies reported that *A. chinense* and its representative active components (such as anabasine and salicin) relieved arthritis, exhibited anti-inflammatory activity and ameliorated rheumatoid arthritis. These abilities are associated with the attenuated expression of proinflammatory cytokines, the inhibition of NF-κB signaling, the improvement of oxidative stress pathways and the relaxation of skeletal and smooth muscle (Zhang et al., 2013; Zhai et al., 2018; Hu et al., 2020).

*Alangium chinense* was documented as a famous toxic herb in traditional Chinese medicine (TCM). Although the therapeutic actions of *A. chinense* have been reported in both clinics and experiments, its clinical application is limited due to its obvious adverse effects on the central nervous system, lungs, liver and smooth muscles (Hu et al., 2020; Meng et al., 2021). Previous clinical studies reported that the oral administration of more than 40 g of *A. chinense* led to severe neurotoxicity, including dizziness, convulsions, muscle weakness and respiratory depression (Zhang et al., 2008). According to the *Dictionary of Chinese Materia Medica*, the 50% lethal dose (LD_50_) of *A. chinense* water extract in mice after intraperitoneal injection was 9.98 g/kg (Zhao et al., 2006). In one study, an intravenous injection of 1.25 g/kg and 4 g/kg *A chinense* water extract resulted in significant toxicity, including respiratory paralysis and muscle weakness in rabbits and beagles, respectively (Zhang et al., 2019). After exposure to intravenous total alkaloids of *A. chinense*, the minimal dose for muscle relaxation and the minimal lethal dose in rabbits were respectively found to be 2.47 mg/kg and 5.65 mg/kg (Zhao et al., 2006). Another study reported that neurotoxicity and hepatoxicity were observed in rabbits following the intravenous administration of total alkaloids of *A. chinense* at a repeated dose of 1.9 mg/kg for 15 days (Zhao et al., 2006). Anabasine, a bioactive chemical, is known as the primary constituent responsible for the therapeutic actions and adverse effects of *A. chinense* (Hu et al., 2020; Meng et al., 2021). Following the intravenous administration of anabasine, the minimal doses for muscle relaxation and respiratory paralysis in rabbits were 1.18 mg/kg and 1.47 mg/kg (Zhao et al., 2006).

Collectively, the above results suggest that *A. chinense* and its active component have narrow therapeutic windows, thus creating a significant challenge for clinical application. Compatibility is an essential aspect of TCM medication. According to the compatibility principle of TCM, the combination of toxic herbs with other herbal medicines can generate the expected therapeutic actions but with lower adverse effects (Steven and Yi, 2021; Xiang et al., 2021). Many researchers have reported that the combined application of compatible herbs in various herbal formulations can weaken or eliminate the toxicity of the poisonous TCM based on well-designed toxic evaluations performed both *in vivo* and *in vitro* (Zhang et al., 2018). Jin Gu Lian prescription consists of a total of five types of herbs: *A. chinense* (Lour.) Harms (Cornaceae; Alangii Radix; Ba Jiao Feng in Chinese), *Gaultheria leucocarpa var. yunnanensis* (Franch.) T. Z. Hsu and R. C. Fang (Ericaceae; Gaultheriae Herba; Tou Gu Xiang in Chinese), *Heptapleurum leucanthum* (R.Vig.) Y. F. Deng (Araliaceae; Schefflerae Leucanthae Caulis seu Folium; Han Tao Ye in Chinese), *Sargentodoxa cuneata* (Oliv.) Rehder and E. H. Wilson (Lardizabalaceae; Sargentodoxae Caulis; Da Xue Teng in Chinese), and *Psammosilene tunicoides* W. C. Wu and C. Y. Wu (Caryophyllaceae; Psammosilenes Radix; Jin Tie Suo in Chinese). Previous research investigated the pharmacokinetic characteristics of a representative formula JGL and its core drug pair (*A. chinense and Sargentodoxa cuneate*)*.* These results demonstrated that combination of the core drug pair with other herbs in JGL decreased the absorption and avoided the accumulation of anabasine by reducing maximum plasma concentration (C_max_) and apparent volume of distribution (V_z/F_), which might reduce the possibility of toxicity of anabasine (Zheng et al., 2022). However, the effects of a compatible approach on the neurotoxicity induced by *A. chinense* has yet to be investigated.

In this study, we aimed to investigate the rationality of the compatible application of *A. chinense* with other herbal medicines in JGL formula in terms of attenuation of toxicity. This study was designed to reveal the effect of compatible herbs in JGL on the attenuation of neurotoxicity induced by *A. chinense* and investigate its mechanism by evaluating the amelioration of oxidative damages, the regulation of neurotoxic biomarkers, the alteration of neurotransmitter levels, and modulation of the pharmacokinetic characteristics of active components in *A. chinense.*

## 2 Materials and methods

### 2.1 Drugs and reagents

#### 2.1.1 Crude drugs and extraction

All crude drugs were supplied by Guizhou Yibai Pharmaceutical Co., Ltd. The voucher specimens are deposited in the Laboratory of Quality Control for Traditional Chinese Medicine in the Institute of Chinese Materia Medica, China Academy of Chinese Medical Sciences, and were identified by Professor Lihua Yan from the institute according to the standard of Chinese pharmacopoeia (2020) and the standard of TCM materials and Ethnic medicinal materials in Guizhou Province (2019). Detailed information was shown in Table 1.

**TABLE 1:** The five herbal medicines of Jin-Gu-Lian formula.

Chinese name	Binomial synonym	Scientific name	Family	Origin	Batch number
Ba Jiao Feng	Alangii Radix	*Alangium chinense* (Lour.) Harms	Cornaceae	Henan	YL-17720220302
Tou Gu Xiang	Gaultheriae Herba	*Gaultheria leucocarpa* var. *yunnanensis* (Franch.) T.Z.Hsu and R.C.Fang	Ericaceae	Guizhou	YL-1720220201
Han Tao Ye	Schefflerae Leucanthae Caulis seu Folium	*Heptapleurum leucanthum* (R.Vig.) Y.F.Deng	Araliaceae	Guangdong	YL-1762022070
Da Xue Teng	Sargentodoxae Caulis	*Sargentodoxa cuneata* (Oliv.) Rehder and E.H.Wilson	Lardizabalaceae	Anhui	JYC-20220206
Jin Tie Suo	Psammosilenes Radix	*Psammosilene tunicoides* W.C.Wu and C.Y.Wu	Caryophyllaceae	Yunnan	YL-17820220304

The dried roots of *A. chinense* (Lour.) Harms (Ba Jiao Feng) were soaked and extracted with boiling water (1:10, 1:8 and then 1:8 w/v) for 2 h following filtration with 150 μm strainer. The extraction step was repeated thrice. The merged mixtures were concentrated under the vacuum freeze dryer to prepare powder and the yield of powder was 5.30% (w/w). The preparation of compatible herbs of JGL consisted of 30% *G. leucocarpa var. yunnanensis* (Franch.) T. Z. Hsu and R. C. Fang (Tou Gu Xiang), 35% *H. leucanthum* (R.Vig.) Y. F. Deng (Han Tao Ye), 30% *S. cuneata* (Oliv.) Rehder and E. H. Wilson (Da Xue Teng), 5% *P. tunicoides* W. C. Wu and C. Y. Wu (Jin Tie Suo). These compatible herbs were prepared through the same procedure and the yield was 5.64% (w/w). The powder of *A. chinense* extract (AC) and the extract of compatible herbs (CH) was analyzed by ultra-performance liquid chromatography-quadrupole-time-of-flight tandem mass (UPLC-Q-TOF-MS) analysis. Anabasine and venoterpine were quantified in AC and the combination of AC and CH. The content of anabasine and venoterpine in AC was 249.2 ± 2.2 μg/g and 2.1 ± 0.0 μg/g, respectively. The content of anabasine and venoterpine in the combination of AC and CH was 175.0 ± 0.9 μg/g and 1.4 ± 0.0 μg/g respectively.

The UPLC-Q-TOF-MS analysis was performed on a Waters ACQUITY UPLC H-Class system with a Waters UPLC BEH C_18_ column (2.1 mm × 100 mm, 1.7 μm). Briefly, the powder of AC or CH was dissolved in water (1:20, w/v), placed in an ultrasonic bath for 30 min, and centrifuged for 5 min at 10,000 rpm. The supernatant was filtered through a 0.22 μm filter membrane for subsequent UPLC-MS analysis. Linear gradient elution with mobile phases of phase A containing the aqueous phases of 0.1% formic acid and phase B containing acetonitrile was applied (0–12 min, 5%–55% B; 12–15 min, 55%–95% B) at a flow rate of 0.3 mL/min. The column was maintained at 35°C and the injection volume was 1 μL. MS analysis was carried out on a Q-TOF high-resolution mass spectrometer (Xevo G2-S; Waters) with an ESI Source. The MS conditions were capillary voltage: 2.2 kV (ESI+), 2.0 kV (ESI-); drying gas (N_2_) flow rate: 600 L/h; drying gas temperature: 400°C; cone voltage: 40 V; ion source temperature: 120°C; scanning mode: positive and negative ion switching scanning; scan range: 100.0–1500.0 m*/z*; collision voltage: 20–50 eV. Data analysis was performed using MassLynx 4.1 software (Waters).

#### 2.1.2 Reagents and chemicals

DL-anabasine (ANA, lot no: C10954677, purity: 97.0%) was obtained from Shanghai Macklin Biochemical Co., Ltd. (Shanghai, China). Venoterpine (VEN, lot no: X31O11L129589, purity: 98.0%) was purchased from Shanghai Yuanye Bio-Technology Co., Ltd. (Shanghai, China). Nicotine (NIC, lot no: V-942900-NU1, purity: 100.0%) was obtained from CFW Laboratories Inc. (Walnut, United States) as internal standard (IS). Chromatographic methanol and acetonitrile were provided by Merck (Darmstadt, Germany), and chromatographic formic acid was provided by ROE SCIENTIFIC (Newark, United States). Other reagents were commercially available and were analytically and chromatographically pure.

### 2.2 Animals, drug administration, and sample collection

**FIGURE 1:**
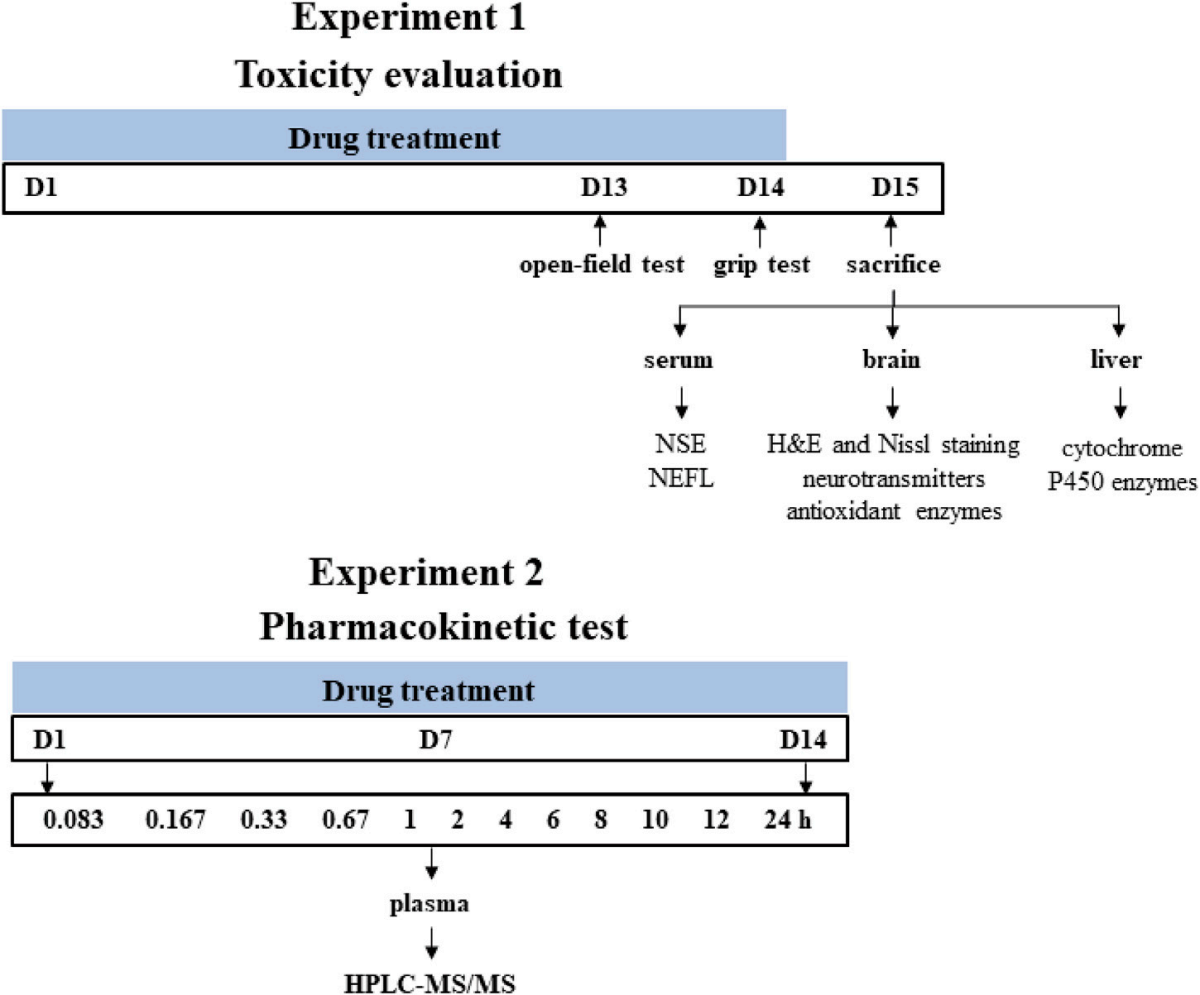
Schematic diagram of the experiment design. Time points represent the days of drug treatment.

All animals were obtained from Beijing Vital Laboratory Animal Technology (Beijing, China) and maintained in a specific pathogen-free laboratory of the Institute of Chinese Materia Medica, China Academy of Chinese Medical Sciences (Beijing, China) under controlled conditions (25°C ± 1°C, 45% relative humidity and a 12 h light/dark cycle). The study protocol was approved by the Institutional Animal Ethical and Welfare Committee (Approval Number: 2021B094, 2021B196). All animal procedures were in accordance with Institutional Animal Care and Use Committee guidelines. A schematic study diagram is shown in Figure 1. The detailed designs of animal experiments were shown as follows.

#### 2.2.1 Experiment 1 (toxicity evaluation)

Male Sprague-Dawley rats (200 ± 20 g) were randomly divided into four groups with eight rats in each group and orally gavaged with the distilled water (control group), *A. chinense* extract (AC group, equivalent to 30 g/kg of crude drugs), extract of compatible herbs of JGL (CH group, equivalent to 30 g/kg of crude drugs), combination of AC with CH (AC/CH group, equivalent to 30 g/kg of crude drugs of *A. chinense* herbs and 30 g/kg of crude drugs of compatible herbs) once-daily for 14 days. One hour after administration on the 13th day (Day 13), the locomotor activity of rats was measured using the open-field test. One hour after the last administration (Day 14), grip force of rats was measured by grip test. On Day 15, rats were anesthetized with pentobarbital sodium (48 mg/kg, i.p.). The blood, brain and liver were collected for further analysis.

#### 2.2.2 Experiment 2 (pharmacokinetic study)

Male Sprague-Dawley rats (220 ± 20 g) were randomly divided into two groups with five rats in each group and orally gavaged with continuous administration (once-daily for 14 days) of *A. chinense* extract (AC, equivalent to 30 g/kg of crude drugs) and continuous administration (once-daily for 14 days) of combination of AC and CH (equivalent to 30 g/kg of crude drugs of *A. chinense* herbs and 30 g/kg of crude drugs of compatible herbs). All rats were fasted (free access to water) for at least 12 h prior to first and last drug administration. Rats were anesthetized with isoflurane for about 30 s and then blood samples were collected at different time after the first and last administration for the two groups. All rats were euthanized with carbon dioxide after the experiment.

### 2.3 Behavioral test

The open field test and grip test were undertaken to investigate the neurobehavioral disorders of rats.

#### 2.3.1 Open-field test

The locomotor activity of animal was measured using the open-field test. After administration for 13 days, rats were placed in a black square chamber (100 cm length × 100 cm width × 50 cm height) and allowed to explore freely for 5 min. The spontaneous movements of rats were recorded by a video camera and analyzed by SMART Video Tracking System V3.0 (Panlab, United States). The parameters recorded included total distance traveled, number of rearing (animal’s double forelimbs leave the ground at the same time, or the two forelimbs are placed on the wall of the cage), total movement time and total rest time.

#### 2.3.2 Grip test

The grip strength meter (YLS-13A, Jinan Yiyan technology development Co. Ltd, China) was used to assess the forelimb grip strength of rats according to manufacturer instruction. After 14 days, rats were gently placed on the grid plate and allowed to grip the metal bars with their front paws. The rats were pulled by the tail slowly. The maximal grip force during the process of steady grasping until losing strength was measured in grams. Each rat was tested thrice.

### 2.4 Histological and pathological assessment

Rats were anesthetized with pentobarbital sodium (48 mg/kg, i.p.). The brain was collected and the left part of brain was fixed in 4% paraformaldehyde in PBS and embedded in paraffin. After locating inferior colliculi, optic chiasma and hypothalamus, the brain was cut transversely to different sections. 4 μm thick slices were cut and stained with hematoxylin and eosin (H&E staining) and toluidine blue (Nissl staining).

### 2.5 ELISA for neuron-specific enolase (NSE) and neurofilament light chain (NEFL) levels

Rats were anesthetized with pentobarbital sodium (48 mg/kg, i.p.). Blood was collected from the abdominal aorta. Serum was separated by centrifugation at 3500 × g for 20 min at room temperature. The levels of NSE and NEFL in serum were measured by rat ELISA kits according to the manufacture instructions (Cusabio, China). The OD was measured at 450/650 nm.

### 2.6 Antioxidant enzymes analysis of brain

After killing the anesthetized rats, brains were collected rapidly, frozen in liquid nitrogen and stored at −80°C until use. The brains were homogenized in nine volumes of 0.9% NaCl with two metal beads at a speed of 60 Hz for 30 s and centrifuged (3500 rpm/min) at 4°C for 15 min. The supernatant was collected. Superoxide dismutase (SOD), glutathione peroxidase (GSH-Px) activities and total antioxidant capacity (T-AOC) were determined using commercial assay kits (Nanjing Jiancheng Bioengineering Institute, China) according to the manufacturer instructions.

### 2.7 LC-MS/MS for neurotransmitter level in the brain

Monoamine and acetylcholine neurotransmitters were analyzed as reported previously with slight modification (Wang L. S. et al., 2019). Briefly, brain tissue was precisely weighed and homogenized in distilled water at a final concentration of 5 g/mL 50 μL brain homogenate was mixed with 10 μL trifluoroacetic acid under the fume hood to precipitate protein. The mixture was centrifuged at 20,000 *g* for 15 min at 4°C. The supernatant was collected for analyzing acetylcholine (Ach), dopamine (DA), 3,4-dihydroxyphenylacetic acid (DOPAC), homovanillic acid (HVA), norepinephrine (NE), and serotonin (5-HT) in the LC-MS/MS system (Agilent, United States).

### 2.8 Pharmacokinetic test

#### 2.8.1 Plasma sample preparation

Blood samples (no more than 150 μL) were collected from the eye venous plexus into tubes containing heparin sodium at 5, 10, 20, 40 min, and 1, 2, 4, 6, 8, 10, 12, 24 h after first and last administration. Plasma was obtained by centrifugation at 12,000 rpm for 3 min at 4 C and stored at −80 C until analysis.

All plasma samples were prepared *via* protein precipitation. An aliquot of 50 μL plasma sample was spiked with 400 μL of dichloromethane containing 10 ng/mL of NIC. After vortexed for 1 min and ultrasound for 1 min, the suspension was centrifuged at 14,000 rpm for 5 min at 4 C. 350 μL of lower solution was transferred to a new tube, and was centrifuged and concentrated at 1,700 rpm and 45 C to dryness. The residue was redissolved in 100 μL of methanol followed by a 1 min vortex and a 2 min ultrasound. The supernatant was injected into the ExionLC high-performance liquid chromatography tandem with a Qtrap 5500 mass spectrometer (HPLC-MS/MS, AB SCIEX, Toronto, Canada) system for analysis after centrifuging at 14,000 rpm for 5 min at 4°C.

#### 2.8.2 HPLC-MS/MS analytical conditions

The concentration of analytes in rat plasma samples was determined by HPLC-MS/MS method. In previous study, an UPLC-MS/MS method was established for simultaneous determination of salidroside, anabasine, chlorogenic acid, and protocatechuic acid in rat plasma and compared the pharmacokinetic properties of two groups of rats after being orally administrated with Jin-Gu-Lian formula and its core drug pair, respectively (Zheng et al., 2022). In our study, it was necessary to investigate the plasma concentrations of anabasine and venoterpine in plasma at the toxic dose, which was higher than that use in Zheng’s study. Therefore, we established an HPLC-MS/MS method for simultaneous determination of the two compounents in plasma of rats, and carried out methodological verification including dilution reliability (detailed information in Supplementary Materials).

The Phenomenex Kinetex C_18_ 100 A LC Column (100 mm × 3 mm, 2.6 μm) was used at a temperature of 40 C. The autosampler temperature was 6 C. After 5 μL injection, mobile phase A (water with 0.05% formic acid) and B (methanol) were used for elution at a flow rate of 0.3 mL/min according to the following procedure: 0–1.0 min, 95% A; 1.0–2.0 min, 95%–5% A; 2.0–3.0 min, 5% A; 3.0–3.1 min, 5%–95% A; 3.1–5.0 min, 95% A.

Multiple reaction monitoring (MRM) scans were used to detect the analytes and IS in the positive ion mode of the electrospray ionization (ESI) source. The ion spray voltage was maintained at 5500 V and curtain gas pressure was maintained at 35 psi. The collision gas was set to six psi and the turbo spray temperature was maintained at 500°C. The nebulizer gas (gas 1) and heater gas (gas 2) were both set at 45 psi, using nitrogen. The precursor/product pairs of ANA, VEN, and IS were *m/z* 163.0→118.1, m*/z* 150.1→132.1, and *m/z* 163.2→130.1, respectively. The declustering potentials (DP) of ANA, VEN, and IS were 74.0, 78.0, and 70.1 V, respectively. The collision energies (CE) for ANA, VEN, and IS were 30.0, 27.0, and 27.2 V, respectively. The dwell time and collision exit potential (CXP) for all compounds were 100 ms and 13 V, respectively.

### 2.9 Analysis of the mRNA levels of cytochrome P450 in the liver

After killing the anesthetized rats, livers were collected, frozen in liquid nitrogen and store in −80°C until use. Total RNA in liver tissues was isolated by the RNA Easy Fast Tissue/Cell Kit (Tiagen, China). Complementary (c)DNA was prepared from 500 ng RNA using the EasyScript^®^ First-Strand cDNA Synthesis SuperMix Kit (TransGen Biotech, China). cDNA was subjected to RT-qPCR to quantify gene expression using PerfectStart Green qPCR SuperMix (TransGen Biotech, China). The primer pairs (Invitrogen, Thermo Fisher Scientific, United States) used for analyses are listed in Supplementary Table S1. Beta-actin was normalized for the amounts of cDNA. Relative expression of target genes was calculated using the 2^−ΔΔCT^ method.

### 2.10 Statistical analysis

Data acquisition and analysis for the concentration of ANA and VEN were conducted using the Analyst 1.7 software (AB SCIEX, Toronto, Canada). Pharmacokinetic parameters of ANA and VEN were calculated using a non-compartment model with the MaS Studio 1.5.2.14 stable software (Shanghai BioGuider Medicinal Technology Co., Ltd, Shanghai, China).

Data were expressed as mean ± standard deviation. Statistical analyses were performed using GraphPad Prism 8.0 software (La Jolla, CA, United States). All data involving toxicity were analyzed using one-way ANOVA followed by a Tukey’s (SOD, GSH-Px, Ach, 5-HT, HVA, open-field test) or Dunnett’s T3 *post hoc* test (NEFL, NE, DA), or Kruskal-Wallis test followed by a Dunn’*s post hoc* test for multiple comparisons (NSE, T-AOC, DOPAC, grip force). The statistical differences involving pharmacokinetic study were performed by independent t-tests. In each case, *p*-value of <0.05 was considered statistically significant.

## 3 Results

### 3.1 Component analysis of AC and CH extracts by UPLC-Q-TOF-MS

**FIGURE 2:**
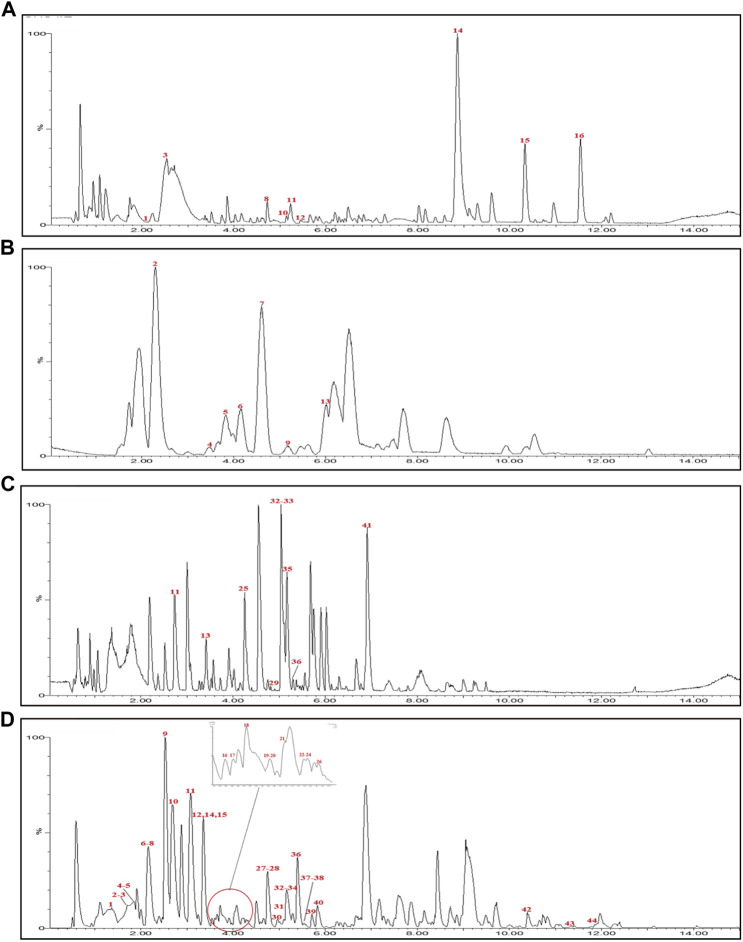
Base peak intensity (BPI) chromatograms for Alangium chinense extract (AC) and extract of compatible herbs of Jin-Gu-Lian formula (CH) by ultra-performance liquid chromatography-quadrupole-time-of-flight tandem mass (UPLC-Q-TOF-MS). **(A)** BPI chromatogram of the positive (ESI+) ionization modes for AC; **(B)** BPI chromatogram of the negative (ESI−) ionization modes for AC; **(C)** BPI chromatogram of the positive (ESI+) ionization modes for CH; **(D)** BPI chromatogram of the negative (ESI−) ionization modes for CH.

**FIGURE 3:**
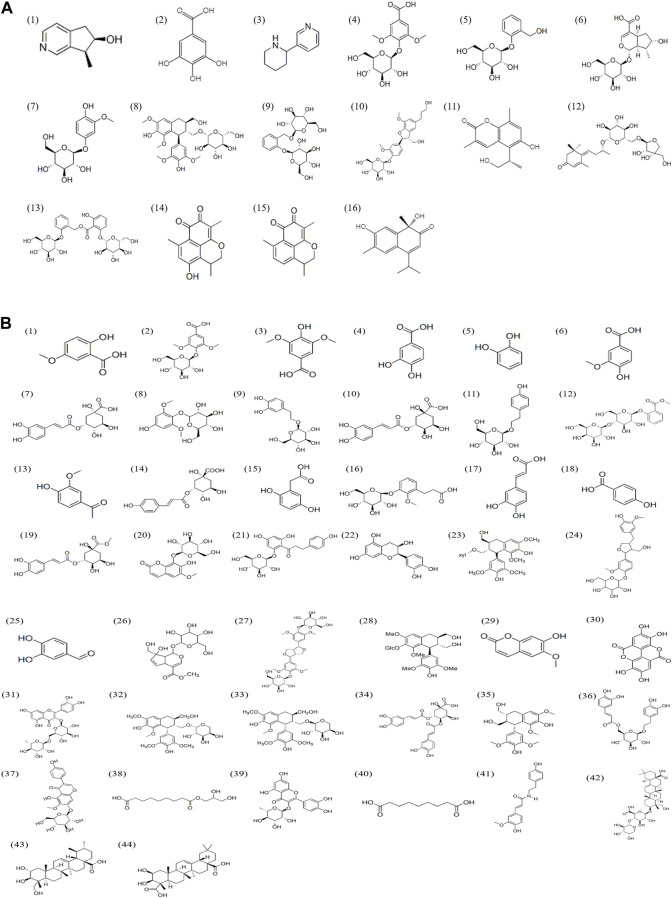
Chemical structures of compounds in the *Alangium chinense* extract **(A)** and the extract of compatible herbs of JGL **(B)**.

UPLC-Q-TOF-MS analysis was performed to identify the constituents of AC and CH extracts. Firstly, literature on the chemical composition of JGL formula have been carefully reviewed and concluded. In this study, the chemical formulae of all compounds were based on high-precision excimer [M + H]^+^, [M + Na]^+^ or [M-H]^-^ with a mass error of 10 ppm and a partial isotopic abundance. Then, the most rational structures were analyzed and identified by searching chemical databases such as Pubchem (https://pubchem.ncbi.nlm.nih.gov/) and Massbank (http://www.massbank.jp). For the isomers, preference would be given to the structures previously reported from the five herbs of JGL formula. Finally, 16 compounds of AC and 44 compounds of CH were confirmed further by fragment ions, retention times, and literature comparison (Table 2; Table 3). The base peak intensity (BPI) chromatograms of AC and CH in negative and positive ionization modes were shown in Figure 2. The chemical structures of the main constitutions found in AC and CH were shown in Figure 3.

**TABLE 2:** The identification of constituents of the *Alangium chinense* extract (AC) by UPLC-Q-TOF-MS.

Peak no.	Name	Formula	Exact m/z	Observed m/z	MS^2^ ions	RT (min)	PPM	Extracting ions	References
1	Venoterpine	C_9_H_11_NO	149.0841	150.0911	108.0440	2.05	−5.3	[M + H]^+^	Zhu et al. (1996)
2	Gallic acid	C_7_H_6_O_5_	170.0215	169.0132	125.0235	2.29	−3.0	[M-H]^-^	Yue (2016)
3	Anabasine	C_10_H_14_N_2_	162.1157	163.1236	146.0959, 134.0964, 120.0814	2.52	0.6	[M + H]^+^	Wei et al. (2020)
4	Glucosyringic acid	C_15_H_20_O_10_	360.1056	359.0971	197.0497, 153.0166	3.53	−1.9	[M-H]^-^	Yue (2016)
5	Salicin	C_13_H_18_O_7_	286.1053	285.0977	191.2886, 123.0449, 121.0283	3.84	1.1	[M-H]^-^	Yue (2016)
6	Loganic acid	C_16_H_24_O_10_	376.1370	375.1286	213.0753, 169.0128, 113.0235	4.15	−1.3	[M-H]^-^	Yue (2016)
7	Tachioside	C_13_H_18_O_8_	302.1002	301.0925	161.0457, 140.0412, 139.0833, 124.0141	4.61	0.7	[M-H]^-^	Yue (2016)
8	(+)-Lyoniresinol 3a-O-*β*-D-glucopyranoside	C_28_H_38_O_13_	582.2312	605.2210	249.1133, 267.1234, 268.1270	4.73	0.0	[M + Na]^+^	Yue (2016)
9	7-O-*β*-Glucopyranosylsalicin	C_19_H_28_O_12_	448.1581	447.1496	285.0017, 123.0069	5.18	−1.6	[M-H]^-^	Zhang et al. (2009)
10	(7S,8R)-Urolignoside	C_26_H_34_O_11_	522.2101	545.2000	249.1131	5.19	0.2	[M + Na]^+^	Yue (2016)
11	(11S)-6-Hydroxy-5-(11-hydroxypropan-12-yl)-3,8-dimethyl-2H-chromen-2-one	C_14_H_16_O_4_	248.1049	249.1130	191.1123, 163.0776	5.23	1.2	[M + H]^+^	Zhang et al. (2015)
12	Chinenionside A	C_24_H_38_O_11_	502.2414	525.2323	371.2097, 209.1543	5.47	2.1	[M + Na]^+^	Yue (2016)
13	Henryoside	C_26_H_32_O_15_	584.1741	583.1697	153.0178, 135.0073, 297.0623, 315.0142, 345.0106	5.91	5.8	[M-H]^-^	Yue (2016)
14	Mansonone H	C_15_H_14_O_4_	258.0892	259.0970	231.1022, 230.0578, 215.0708	8.86	0.0	[M + H]^+^	Zhang et al. (2013)
15	Mansonone E	C_15_H_14_O_3_	242.0943	243.1024	199.0748, 115.0554	10.33	1.2	[M + H]^+^	Zhang et al. (2013)
16	Lacinilene C	C_15_H_18_O_3_	246.1256	247.1334	219.1387, 201.1290	11.54	0.0	[M + H]^+^	Zhang et al. (2013)

**TABLE 3:** The identification of constituents of the extract of compatible herbs of JGL (CH) by UPLC-Q-TOF-MS.

Peak no.	Name	Formula	Exact m/z	Observed m/z	MS^2^ ions	RT (min)	PPM	Extracting ions	References
1	5-Methoxysalicylic acid	C_8_H_8_O_4_	168.0423	167.0344	152.0105, 108.0205	1.36	−1.8	[M-H]^-^	Zhou et al. (2022)
2	Glucosyringic acid	C_15_H_20_O_10_	360.1056	359.0977	197.0448, 182.0211	1.70	−0.3	[M-H]^-^	Liu et al. (2022)
3	Syringic acid	C_9_H_10_O_5_	198.0528	197.0446	182.0215, 166.9987, 138.0311, 123.0080	1.70	−2.0	[M-H]^-^	Liu et al. (2022)
4	Protocatechuic acid	C_7_H_6_O_4_	154.0266	153.0189	135.0432, 109.0282	1.85	0.7	[M-H]^-^	Liu et al. (2022)
5	Catechol	C_6_H_6_O_2_	110.0368	109.0288	-	1.85	−1.8	[M-H]^-^	Zhou et al. (2022)
6	Vanillic acid	C_8_H_8_O_4_	168.0423	167.0342	152.0103, 123.0480	2.12	−1.2	[M-H]^-^	Liu et al. (2022)
7	Neochlorogenic acid	C_16_H_18_O_9_	354.0951	353.0871	191.0559, 179.0345, 135.0445	2.15	−0.6	[M-H]^-^	Wang X. et al. (2019)
8	4-Hydroxy-2,6-dimethoxyphenol-1-O-β-D-glucopyranoside	C_14_H_20_O_9_	332.1107	331.1028	256.0684, 169.0497	2.25	−0.3	[M-H]^-^	Wang X. et al. (2019)
9	3,4-Dihydroxyphenylethyl alcohol glucoside	C_14_H_20_O_8_	316.1158	315.1085	135.0445	2.52	1.6	[M-H]^-^	Liu et al. (2022)
10	Chlorogenic acid	C_16_H_18_O_9_	354.0951	353.0872	191.0558, 179.0344, 161.0240, 127.0394, 109.0289	2.69	8.0	[M-H]^-^	Liu et al. (2022)
11	Salidroside	C_14_H_20_O_7_	300.1209	299.1135	179.0343, 161.0236, 137.0233, 113.0233	3.06	1.3	[M-H]^-^	Liu et al. (2022)
12	Methyl salicylate lactoside/methylsalicylate gentiobioside	C_20_H_28_O_13_	476.1530	475.1453	443.1357, 281.0682, 151.0396, 137.0226	3.31	0.2	[M-H]^-^	Wang X. et al. (2019)
13	Apocynin	C_9_H_10_O_3_	166.0630	167.0705	149.0227, 107.0464	3.38	−1.8	[M + H]^+^	Liu et al. (2022)
14	5-O-p-coumaroyl quinic acid	C_16_H_18_O_8_	338.1002	337.0918	191.0549, 163.0389	3.43	−1.5	[M-H]^-^	Wang X. et al. (2019)
15	Homogentisic acid	C_8_H_8_O_4_	168.0423	167.0341	149.0446, 139.0397, 109.0294	3.43	−1.8	[M-H]^-^	Zhou et al. (2022)
16	3-[3-(*β*-D-Glucopyranosyloxy)-2-methoxyphenyl]propanoic acid	C_16_H_22_O_9_	358.1264	357.1179	269.0680, 195.0646	3.56	−2.2	[M-H]^-^	Zhou et al. (2022)
17	Caffeic acid	C_9_H_8_O_4_	180.0423	179.0338	135.0440	3.61	−3.4	[M-H]^-^	Zhou et al. (2022)
18	4-Hydroxybenzoic acid	C_7_H_6_O_3_	138.0317	137.0238	-	3.76	−0.7	[M-H]^-^	Wang X. et al. (2019)
19	Methyl chlorogenate	C_17_H_20_O_9_	368.1107	367.1031	179.0564, 161.0236, 135.0434, 134.0366	3.88	0.5	[M-H]^-^	Liu et al. (2022)
20	Fraxin	C_16_H_18_O_10_	370.0900	369.0823	207.0287, 192.0053	3.88	0.3	[M-H]^-^	Zhou et al. (2022)
21	Phloridzin	C_21_H_24_O_10_	436.1369	435.1291	273.0740	4.00	1.1	[M-H]^-^	Zhou et al. (2022)
22	(−)-Epicatechin	C_15_H_14_O_6_	290.0790	289.0712	179.0333, 165.0550, 137.0235, 125.0232	4.21	0.0	[M-H]^-^	Liu et al. (2022)
23	(−)-5′-Methoxyisolariciresinol-2α-O-*β*-D-xylopyranoside	C_26_H_34_O_11_	522.2011	521.2018	506.1494, 359.1494, 341.0912	4.22	−1.0	[M-H]^-^	Wang X. et al. (2019)
24	Lariciresinol 4-O-glucoside	C_26_H_34_O_11_	522.2101	521.2029	359.1494, 344.1256, 329.1039, 313.1070	4.22	−1.0	[M-H]^-^	Zhou et al. (2022)
25	3,4-Dihydroxybenzaldehyde	C_7_H_6_O_3_	138.0317	139.0394	111.0438	4.28	−0.7	[M + H]^+^	Zhou et al. (2022)
26	Methyl 1-(hexopyranosyloxy)-7-hydroxy-7-(hydroxymethyl)-1,4a,7,7a-tetrahydrocyclopenta [c]pyran-4-carboxylate	C_17_H_24_O_11_	404.1319	403.1247	371.0871, 327.1069, 241.0736, 223.0630, 191.0561, 179.0360, 165.0556, 139.0397	4.32	1.2	[M-H]^-^	Zhou et al. (2022)
27	Liriodendrin	C_34_H_46_O_18_	742.2684	741.2606	417.1549, 387.1077, 181.0499, 166.0264	4.75	0.1	[M-H]^-^	Zhou et al. (2022)
28	(+)-Lyoniresin-4-yl-β-D-glucopyranoside	C_28_H_38_O_13_	582.2217	581.2249	419.1671, 404.1458, 233.0809	4.79	2.6	[M-H]^-^	Liu et al. (2022)
29	Scopoletin	C_10_H_8_O_4_	192.0423	193.0498	165.0526, 149.0241	4.90	−1.6	[M + H]^+^	Zhou et al. (2022)
30	Ellagic acid	C_14_H_6_O_8_	302.0063	300.9992	283.0592, 229.0130	5.00	1.3	[M-H]^-^	Zhou et al. (2022)
31	Rutin	C_27_H_30_O_16_	610.1534	609.1456	300.0276, 271.0266, 255.0281, 179.0340	5.06	4.1	[M-H]^-^	Zhou et al. (2022)
32	(+)-Lyoniresinol-2-α-O-β-L-arabinopyranoside	C_27_H_36_O_12_	552.2207	551.2149	536.1904, 419.1715, 401.1611, 386.1366	5.16	3.6	[M-H]^-^	Wang X. et al. (2019)
33	Gaultheroside A	C_27_H_36_O_12_	552.2207	551.2129	389.1226	5.16	−0.2	[M-H]^-^	Zhou et al. (2022)
34	Isochlorogenic acid B	C_25_H_24_O_12_	516.1268	515.1198	353.0892, 191.0554, 179.0349, 173.0452, 161.0239, 135.0443	5.23	1.4	[M-H]^-^	Zhou et al. (2022)
35	(+)-Lyoniresinol	C_22_H_28_O_8_	420.1784	419.1703	404.1461, 373.1283, 389.1586	5.30	−0.7	[M-H]^-^	Wang X. et al. (2019)
36	Calceolarioside B	C_23_H_26_O_11_	478.1475	477.1397	315.1084, 179.0343, 161.0240, 135.0439	5.40	0.2	[M-H]^-^	Zhou et al. (2022)
37	Tectoridin	C_22_H_22_O_11_	462.1162	461.1084	315.0719	5.55	5.0	[M-H]^-^	Zhou et al. (2022)
38	9-(2,3-dihydroxypropoxy)-9-oxononanoic acid	C_12_H_22_O_6_	262.1416	261.1338	187.1004, 125.0235	5.61	1.5	[M-H]^-^	Zhou et al. (2022)
39	Quercitrin	C_21_H_20_O_11_	448.1006	447.0927	300.0267, 271.0249, 243.0244	5.71	0.9	[M-H]^-^	Zhou et al. (2022)
40	Azelaic acid	C_9_H_16_O_4_	188.1049	187.0970	143.0343	5.86	0.0	[M-H]^-^	Zhou et al. (2022)
41	Feruloyltyramine	C_18_H_19_NO_4_	313.1314	314.1390	177.0550, 145.0290, 121.0650, 117.0338	6.91	−0.6	[M + H]^+^	Zhou et al. (2022)
42	scheffleside K	C_41_H_64_O_14_	780.4296	779.4228	629.3684, 471.3461	10.39	1.3	[M-H]^-^	Wang et al. (2014)
43	Asiatic acid	C_30_H_48_O_5_	488.3502	487.3418	469.3308, 391.0167	11.31	−1.0	[M-H]^-^	Liu et al. (2022)
44	Medicagenic acid	C_30_H_46_O_6_	502.3294	501.3216	483.2984, 439.3230	11.86	0.8	[M-H]^-^	Zhou et al. (2022)

### 3.2 Behavioral changes in rats

The neurotoxic effects were evaluated by detecting neurobehavioral disorders using the open field test and grip test. In the open field test (Table 4), AC treatment significantly inhibited locomotor activity when compared to the control rat, as evidenced by the reduced total distance traveled, the total movement time, the number of rearing and the increased total rest time. This effect was partially prevented by treatment with a combination of AC and CH (*p* < 0.05). Similar results were observed in the grip test. As shown in Table 4, the mean forelimb grip strength in AC-treated rats (918.2 ± 50.3 g) was markedly lower than that of control rats (1206.0 ± 107.0 g) (*p* < 0.01). Rats administered with a combination of AC and CH had significantly increased grip strength (1192.0 ± 55.5 g) when compared to only AC-treated rats (*p* < 0.01). These results suggested that the compatible herbs of JGL could mitigate the neurobehavioral abnormalities induced by *A. chinense.*

**TABLE 4:** Effect of *Alangium chinense* and the compatible herbs on the behavioral activity in rats.

Groups	Open-field test				Grip force(g)
	Total distance traveled (cm)	Total movement time(s)	Total rest time(s)	Number of rearing	
Control	3798.65 ± 419.15	258.69 ± 9.44	41.31 ± 9.44	29 ± 6	1206.19 ± 106.95
AC	2436.09 ± 485.58^##^	226.62 ± 5.87^##^	73.38 ± 5.87^##^	18 ± 6^#^	918.18 ± 50.27^##^
CH	3317.65 ± 786.39	245.17 ± 9.04	54.83 ± 9.04	29 ± 10	1156.34 ± 32.11
AC/CH	3354.07 ± 500.33*	250.17 ± 18.76*	49.83 ± 18.76*	31 ± 5*	1191.89 ± 55.53**

Quantitative data represent the mean ± SD, from 6–8 rats in each group. ^#^ *p* < 0.05, ^##^ *p* < 0.01, compared with control. *p < 0.05, **p < 0.01, compared with only AC treatment.

### 3.3 Neuronal morphology of the rat brain

**FIGURE 4:**
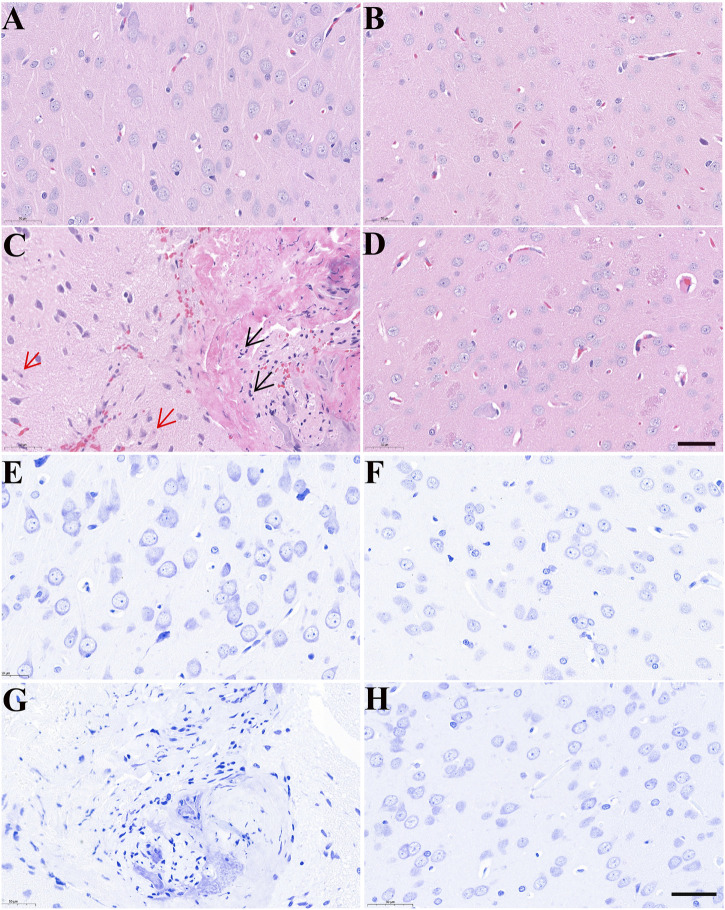
The effects of *Alangium chinense* combined with the compatible herbs of JGL on the morphological structure of neurons in the brain. Rats were orally gavaged with distilled water (control group, A and E), extract of compatible herbs of JGL (CH group, B and F), *Alangium chinense* extract (AC group, C and G), a combination of AC and CH (AC/CH group, D and H) once-daily for 14 days. Scale bar = 50 μm in H&E **(A–D)** and Nissl staining **(E–H)**. Black arrows indicated nucleus pycnosis, karyorrhexis and neuronal necrosis. Red arrows indicated neuronal eosinophilic degeneration.

Morphological changes in brain tissues were detected by H&E and Nissl staining. As shown in Figure 4, the structure of neurons in control rats was normal with regular, round or oval nuclei and nucleoli, and clear Nissl bodies in the cytoplasm. After treatment with AC, there was clear cellular damage in the ventral striatum, as evidenced by neuronal eosinophilic degeneration, nucleus pycnosis, karyorrhexis, neuronal necrosis, reduced Nissl bodies and scattered arrangement. Compared to only AC treatment, except slight neuronal eosinophilic degeneration, we almost did not observe these aforementioned morphological properties in the brain of rats treated with AC and CH.

### 3.4 Serum levels of neurotoxicity biomarkers

**FIGURE 5:**
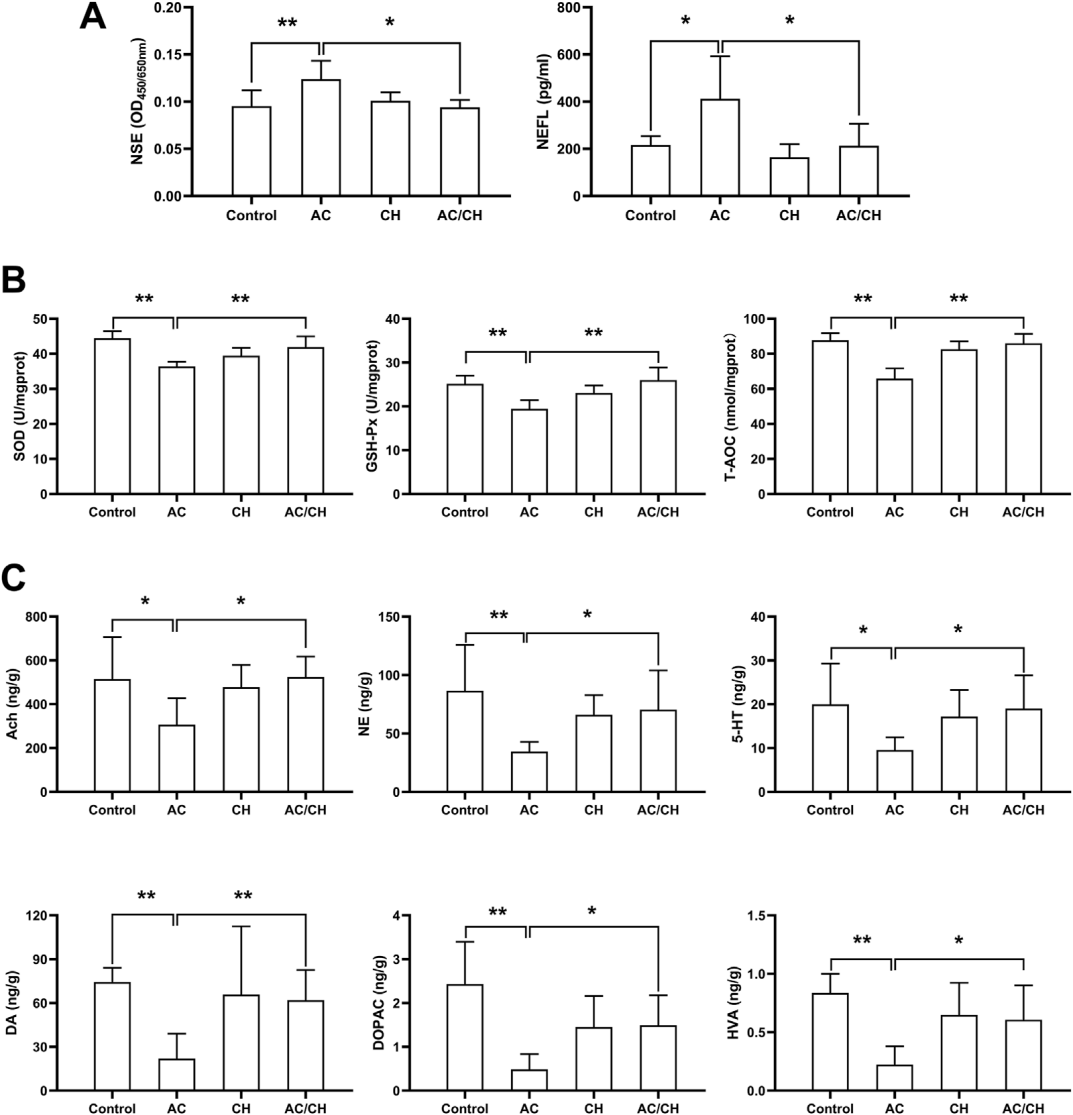
The effects of *Alangium chinense* combined with the compatible herbs of JGL on neurotoxicity biomarkers, oxidative stress and neurotransmitters. Rats were orally gavaged with distilled water (control group), *Alangium chinense* extract (AC group), extract of compatible herbs of JGL (CH group) or a combination of AC and CH (AC/CH group) once-daily for 14 days. **(A)** Serum NSE and NEFL levels were analyzed by ELISA assays. **(B)** Superoxide dismutase (SOD) activity, glutathione peroxidase (GSH-Px) activity and total antioxidant capacity (T-AOC) in brain were detected. **(C)** The levels of monoamine and acetylcholine neurotransmitters in brain were analyzed by LC-MS/MS. Quantitative data represent the mean ± SD from 6–8 rats in each group. *p < 0.05, **p < 0.01.

Given that CH can ameliorate AC-induced neurobehavioral disorders in rats, we investigated the effects of AC in absence or presence of CH on neurotoxic biomarkers. As shown in Figure 5A, compared with control rats, AC only treatment significantly increased the serum levels of NSE and NEFL (*p* < 0.05, *p* < 0.01). Compared with AC-treated rats, the combined AC and CH treatment resulted in a significant reduction of NSE and NEFL levels (*p* < 0.05).

### 3.5 Oxidative stress biomarkers in rat brains

Oxidative damage has been reported to be an essential mechanism of neurotoxicity. Here, we investigated the levels of antioxidants in brain tissues (Figure 5B). AC treatment considerably reduced the activity of SOD, GSH-Px and T-AOC in rat brains when compared to controls (*p* < 0.01), thus indicating serious oxidative damage. Compared with AC treatment, the combined AC and CH treatment significantly enhanced the activity of SOD, GSH-Px and T-AOC in rat brains (*p* < 0.01). These data suggested that the compatible herbs of JGL could reduce oxidative damage in brain induced by *A. chinense*.

### 3.6 Neurotransmitter levels in rat brains

Next, we determined the levels of neurotransmitters in rat brains. As shown in Figure 5C, the levels of some monoamine and acetylcholine neurotransmitters reduced significantly in brains of AC-treated rats when compared to control rats, including Ach, DA, DOPAC, HVA, NE, and 5-HT (*p* < 0.05, *p* < 0.01). Compared with AC treatment, combined AC and CH treatment led to a significant increase in all six neurotransmitters (*p* < 0.05).

### 3.7 Pharmacokinetics of ANA and VEN in rat

**FIGURE 6:**
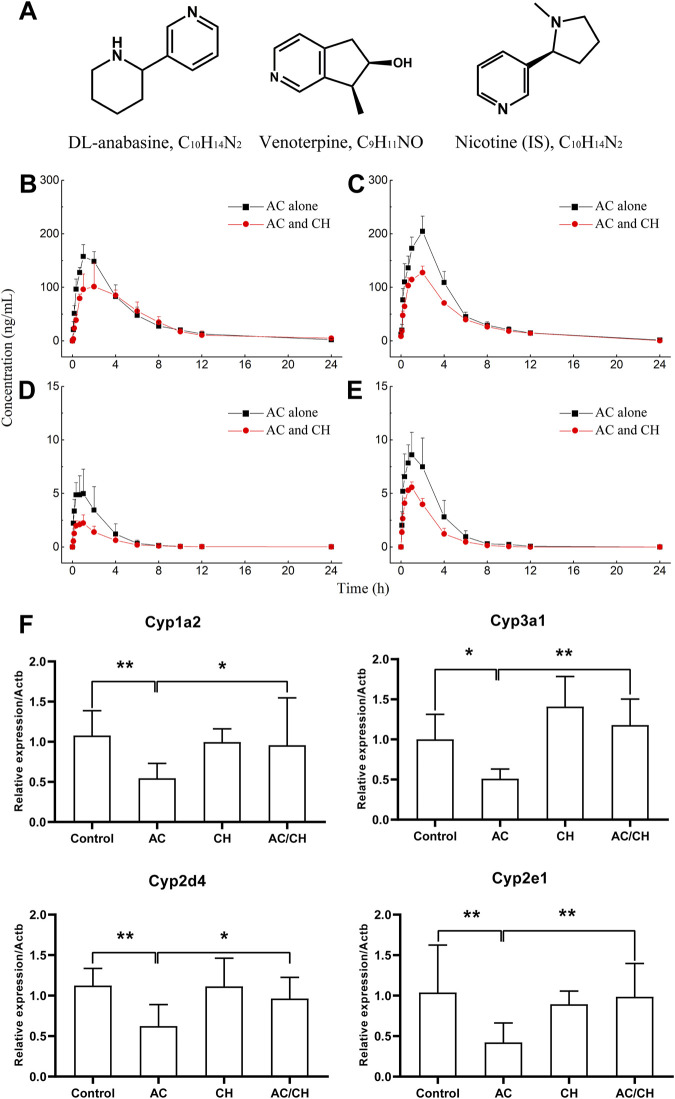
Pharmacokinetic process for the active components of *Alangium chinense* and the gene expression of hepatic cytochrome P450. **(A)** Chemical structure of DL-anabasine (ANA), venoterpin (VEN) and nicotine (IS). In a pharmacokinetic study **(B–E)**, male rats were treated with AC alone or co-administration of AC and CH once a day for 14 days. Mean rat plasma concentration-time profiles of ANA **(B)** and VEN **(D)** after single administration, ANA **(C)** and VEN **(E)** after continuous administration. Data are expressed as mean ± SD (*n* = 5). The gene expression of cytochrome P450 isozymes in liver was analyzed by RT-qPCR **(F)**. The relative expression of target genes was calculated using the 2^−ΔΔCT^ method. The amount of each transcript was normalized to that of beta-actin. Quantitative data represent the mean ± SD of eight rats. *p < 0.05, **p < 0.01.

Anabasine (ANA) and venoterpine (VEN) have been reported to exhibit pharmacological effects and are the major active components in *A. chinense* (Zhu et al., 1996; Hu et al., 2020). Therefore, we selected ANA and VEN as marker compounds to detect the effect of compatible herbs in JGL on the pharmacokinetics of *A. chinense*. The chemical structures of the two analytes (ANA and VEN) and IS are shown in Figure 6A. The HPLC-MS/MS method was validated as described in Supplementary Materials and the results were shown in Supplementary Figure S1; Supplementary Tables S2–S6.

#### 3.7.1 The pharmacokinetics of ANA and VEN in rats after single administration

The single administration of AC and the combination of AC/CH led to ANA and VEN entering the blood within 5 min (Figures 6B,D). The concentrations of ANA and VEN in plasma at different timepoints are shown in Supplementary Table S7. Table 5 shows the main pharmacokinetic parameters, as calculated by the non-compartment model.

**TABLE 5:** The pharmacokinetic parameters of anabasine (ANA) and venoterpin (VEN).

Analytes	PK parameters	Single administration		14 days continuous administration	
		AC	AC and CH	AC	AC and CH
ANA	t_1/2_ (h)	3.08 ± 0.73	4.27 ± 1.11	3.21 ± 0.43	2.55 ± 0.61^#^
	t_max_ (h)	1.20 ± 0.45	1.53 ± 0.65	1.80 ± 0.45	2.00 ± 0.00
	C_max_ (ng/mL)	159.62 ± 19.64	115.79 ± 20.62**	204.71 ± 28.28^#^	127.51 ± 12.09**
	AUC_0-t_ (h·ng/mL)	830.64 ± 131.28	733.71 ± 133.58	1028.67 ± 39.20^#^	746.93 ± 35.00**
	AUC_0-∞_(h·ng/mL)	852.10 ± 121.22	763.78 ± 140.45	1036.87 ± 35.91^#^	748.7 ± 36.50**
	MRT_0-t_ (h)	4.29 ± 0.87	5.43 ± 0.93	4.42 ± 0.36	4.77 ± 0.44
	MRT_0-∞_ (h)	4.72 ± 0.74	6.34 ± 1.20*	4.62 ± 0.40	4.83 ± 0.49^#^
VEN	t_1/2_ (h)	2.45 ± 0.09	3.49 ± 0.76*	1.31 ± 0.26^##^	1.12 ± 0.22^##^
	t_max_ (h)	0.67 ± 0.33	1.07 ± 0.60	0.93 ± 0.15	0.93 ± 0.15
	C_max_ (ng/mL)	5.46 ± 1.80	2.25 ± 0.76**	9.08 ± 1.78^#^	5.67 ± 0.34**^##^
	AUC_0-t_ (h·ng/mL)	15.71 ± 8.21	7.29 ± 2.50	30.40 ± 10.73^#^	16.18 ± 2.82*^##^
	AUC_0-∞_(h·ng/mL)	15.76 ± 8.24	7.47 ± 2.60	30.64 ± 10.88^#^	16.44 ± 2.72*^##^
	MRT_0-t_ (h)	2.27 ± 0.25	3.24 ± 0.49*	2.25 ± 0.41	1.95 ± 0.39^##^
	MRT_0-∞_ (h)	2.34 ± 0.25	3.85 ± 0.99*	2.32 ± 0.43	2.04 ± 0.36^##^

Rats were treated with single administration and 14 days continuous administration of AC, combination of AC, and CH., Quantitative data were the mean ± SD of five rats. *p < 0.05, **p < 0.01, compared with only AC treatment. ^#^ *p* < 0.05, ^##^ *p* < 0.01, compared with single administration.

Compared with the single administration of AC, the combined AC and CH treatment led to a significant decrease in the C_max_ (115.79 ± 20.62 ng/mL) of ANA (*p* < 0.01) and a significant increase in the mean residence time (MRT_0-∞_, 6.34 ± 1.20 h) of ANA (*p* < 0.05). The half time (t_1/2_, 4.27 ± 1.11 h), area under the plasma concentration-time curve (AUC_0-t_, 733.71 ± 133.58 ng·h/mL) and AUC_0-∞_ (763.78 ± 140.45 ng·h/mL) of ANA showed a downward trend, but there was no statistical significance due to large individual differences. There was no significant change in the t_max_ (1.53 ± 0.65 h) and MRT_0-t_ (5.43 ± 0.93 h) of ANA.

Compared with the single administration of AC, the C_max_ (2.25 AUC± 0.76 ng/mL) of VEN decreased significantly (*p* < 0.01), while the t_1/2_ (3.49 ± 0.76 h), MRT_0-t_ (3.24 ± 0.49 h) and MRT_0-∞_ (3.85 ± 0.99 h) of VEN increased significantly (*p* < 0.05, *p* < 0.05, *p* < 0.05) in rats treated with the combination of AC and CH. The AUC_0-t_ (7.29 ± 2.50 ng·h/mL) and AUC_0-∞_ (7.47 ± 2.60 ng·h/mL) of VEN showed a downward trend, but there was no statistical significance due to large individual differences. There was no significant change in t_max_ (1.07 ± 0.60 h).

#### 3.7.2 The pharmacokinetics of ANA and VEN in rats after continuous administration

We also detected the pharmacokinectics of ANA and VEN after 14 days of continuous administration with AC or a combination of AC and CH (Figures 6C,E). The plasma concentrations of ANA and VEN were shown in Supplementary Table S8. The main pharmacokinetic parameters were calculated by using the non-compartment model and were shown in Table 5.

Compared with the continuous administration of AC, the C_max_ (127.51 ± 12.09 ng/mL), AUC_0-t_ (746.93 ± 35.00 ng ·h/mL) and AUC_0-∞_ (748.7 ± 36.50 ng·h/mL) of ANA was decreased significantly (*p* < 0.01, *p* < 0.01, *p* < 0.01) in rats administered continuously with a combination of AC and CH, while the t_1/2_ (2.55 ± 0.61 h), t_max_ (2.00 ± 0.00 h), MRT_0-t_ (4.77 ± 0.44 h) and MRT_0-∞_ (4.83 ± 0.49 h) of ANA did not change significantly. Similar pharmacokinetic characteristics were observed in the main parameters of VEN, as evidenced by a significant decrease in C_max_ (5.67 ± 0.34 ng/mL), AUC_0-t_ (16.18 ± 2.82 ng·h/mL) and AUC_0-∞_ (16.44 ± 2.72 ng·h/mL) (*p* < 0.01, *p* < 0.05, *p* < 0.05, respectively).

### 3.8 mRNA expression of hepatic drug-metabolizing cytochrome P450 enzymes

Cytochrome P450 is the most important metabolic enzyme family in the liver that accounts for either metabolic detoxification or activation in toxicity (Guengerich, 2008). They play key roles in the pharmacokinetic herb-herb interactions (Rittle and Green, 2010). Here, we analyzed the expression of P450 isozymes in rat livers by RT-qPCR. As shown in Figure 6F, AC treatment resulted in the significant downregulation of the gene expression of cytochrome P450 isozymes, including the cytochrome P450 family 1 subfamily a polypeptide 2 (*Cyp1a2*), cytochrome P450 family 3 subfamily a polypeptide 1 (*Cyp3a1*), cytochrome P450 family 2 subfamily d polypeptide 4 (*Cyp2d4*) and cytochrome P450 family 2 subfamily e polypeptide 1 (*Cyp2e1*) (*p* < 0.05; *p* < 0.01). Combined administration of AC and CH significantly reduced the downregulation of the mRNA expression of cytochrome P450 isozymes induced by AC (*p* < 0.05; *p* < 0.01).

## 4 Discussion

Previous studies described *A. chinense* as a ‘double-edged sword’. On one hand, *A. chinense* is used as a medicine for the treatment of rheumatic arthritis, acroanesthesia, and fractures in the Miao population in China (Li et al., 2017). On the other hand, *A. chinense* is famous as a poisonous medicine that results in toxicity involving different organs, such as central nervous system (CNS), lungs, liver and smooth muscles (Hu et al., 2020; Meng et al., 2021). Clinical studies have reported that the oral administration of more than 40 g of *A. chinense* led to severe neurotoxicity, including dizziness, convulsions, muscle weakness and respiratory depression (Zhang et al., 2008). According to the compatibility principle of traditional Chinese medicine, the combination of *A. chinense* with other herbs in the JGL formula could yield the expected therapeutic effects with synergistic action when used to treat rheumatic arthritis but with no severe adverse actions. However, little is known about the effect of compatible herbs in JGL on the neurotoxicity induced by *A. chinense*. In the present study, AC treatment resulted in neurobehavioral disorders, as evidenced by reduced locomotor activity and muscle strength. H&E and Nissl staining showed that AC treatment induced morphological changes in neurons. Previous studies reported that anabasine, the alkaloid of *A. chinense*, led to significant behavioral changes in terms of fetal movement, memory and attention (Levin et al., 2014; Green et al., 2018). In current study, the compatible herbs of JGL could ameliorate the *A. chinense*-induced neurotoxicity by reducing the frequency of induced abnormal behaviors and morphological damage in neurons.

Neuron-specific enolase (NSE) is abundant in neurons of the central nervous system (CNS) and is considered as a neurotoxic marker to assess cerebral neurodegeneration in a wide range of CNS disorders (Sahu et al., 2017). Neurofilaments are released when the axon of a neuron incurs damage. The serum levels of neurofilament light chain (NEFL) can indicate neuronal cell death in the brain and serve as a potential fluid biomarker of neurotoxicity (Sano et al., 2021). We found that AC treatment induced enhanced levels of NSE and NEFL in rat brains, thus concurring with the previous finding that excessive NSE levels are due to oxidative stress (Ciancarelli et al., 2015). The compatible herbs of JGL could reduce the overexpression of NSE and NEFL.

A diverse range of molecular mechanisms are involved in neurotoxicity. Of these, reactive oxygen species (ROS) are known to play an essential role in neuronal fate and development (Wilson et al., 2018). An imbalance between ROS production and the activity of the antioxidant system can lead to excessive ROS accumulation and oxidative stress, thus causing damage to DNA, RNA, protein and lipids (Schieber and Chandel, 2014). In neurocytes, oxidative damage can adversely affect several neuronal functions, including locomotor activity, muscle strength, memory, learning and cognition, thus contributing to developmental neurotoxicity (Spinu et al., 2019). Some antioxidants, including SOD, GSH-Px, catalase (CAT) and glutathione (GSH), can directly and indirectly remove free radicals and ameliorate developmental neurotoxicity (Nishimura et al., 2021). Sesquiterpenes and alkaloids from *A. chinense* were previously shown to exert antioxidant activities against cysteine-induced rat liver microsomal lipid peroxidation (Zhang et al., 2013). Salicin from *A. chinense* can inhibit ROS generation, reduce the levels of malondialdehyde (MDA) and increase the levels of several antioxidant enzymes, including glutathione (GSH), SOD and catalase (CAT) via the Nrf2-HO-1-ROS pathway (Zhai et al., 2018). In agreement with previous reports, we found that AC treatment gave rise to considerable oxidative damage, as evidenced by obvious reductions in the activities of SOD, GSH-Px and T-AOC. The combination of CH with AC could reduce AC-induced oxidative damage by increasing the activity of the cellular antioxidant system. These data suggest that the compatible herbs of JGL could mitigate the neurotoxicity of *A. chinense* by regulating oxidative stress-related disorders.

Neurotransmitters play important roles in the function of the nervous system and mainly include monoamines, amino acids and choline (Vogt, 2019). Neurotransmitter abnormalities are regarded as a biomarker of neurotoxicity and are responsible for numerous neurodegenerative diseases and brain injuries (Iadecola et al., 2001; Sun et al., 2018; Stradtman and Freeman, 2021; Tinkov et al., 2021). Many neurotoxicants are known to exert adverse effects in the concentration and signaling of neurotransmitters (Stradtman and Freeman, 2021; Tinkov et al., 2021). A significant reduction in the levels of monoamine neurotransmitters, including 5-HT, DA, DOPAC and NE was reported to be associated with impaired attention, locomotor dysfunction, disrupted motor coordination, and impaired learning tasks (Sun et al., 2018; Conley et al., 2020; Stradtman and Freeman, 2021). The disruption of cholinergic neurotransmission, as evidenced by upregulated AChE activity, the inhibition of choline acetyltransferase activity, cholinergic neuron death and the reduction of ACh levels is associated with oxidative damage and known to be involved in locomotor and motor deficits (Tinkov et al., 2021). Anabasine, the active component of AC, has been identified as an agonist of nAChRs with greater affinity for α7 nAChR and lower affinity for α4β2 subtypes (Levin et al., 2014). Anabasine-induced α7 nAChR activation results in the release of different transmitters, including dopamine, norepinephrine, serotonin, histamine, GABA and glutamate. These alterations can explain the effect of anabasine on behavioral changes including fetal movement, memory and attention (Levin et al., 2014; Green et al., 2018). Hsieh et al. (1993) previously reported that AC extract affected the motor activity and changed the concentration of monoamine in a rat model. Studies involving another species from the Alangium genus, *Alangium platanifolium*, showed similar effects on neurobehavioral changes as *A. chinense* (Hu et al., 2020). Zhu et al. (1996) reported that *A. platanifolium* extract interacted strongly with a number of neurotransmitters, including α2-adrenoceptor, 5HT1, 5HT2, dopamine 1, dopamine 2, GABA_A_ and GABA_B_ and plays a role in locomotor activity, muscle relaxant activity and analgesic activity. Our present results were in accordance with these previous literatures. The levels of neurotransmitters were illustrated in Figure 5C. AC treatment led to abnormalities in the concentrations and metabolism of neurotransmitters in the brain. Combined treatment with CH and AC reduced the attenuation of monoamine and acetylcholine neurotransmitter levels induced by AC. These observations suggested that the compatible herbs of JGL could regulate the abnormal concentrations and metabolisms of neurotransmitters to mitigate the neurotoxicity induced by *A. chinense*.

The combinational principle of TCM is a primary approach used to reduce the toxicity of toxic herbs by interacting with other herbal materials that cause changes in the pharmacokinetic process, decrease the exposure levels of toxic constituents or inhibit toxic metabolism (Zhang et al., 2018). The toxicity of drugs is directly related to the exposure levels of components *in vivo* and may be positively correlated (Zhang et al., 2018; Chang et al., 2020). In the present study, significant differences of pharmacokinetic profiling in rats were observed between AC treatment and the co-administration of AC and CH. Continuous administration with combination of AC and CH for 14 days demonstrated that the C_max_ of ANA reduced from 204.71 to 127.51 ng/mL, AUC_0-t_ felt down from 1028.67 to 746.93 ng·h/mL and AUC_0-∞_ felt down from 1036.87 to 748.70 ng·h/mL in comparison with AC treatment. The decrease of C_max_ and AUC in response to the combined administration of AC and CH indicated lower absorption and reduced exposure level of ANA in plasma, which might bring about a lower possibility of toxicity induced by AC. Similar results were found for these pharmacokinetic parameters of ANA following the single administration of the extracts. Additionally, single administration of AC and CH led to longer MRT of ANA than AC treatment, which suggested that the compatibility of AC with CH in JGL formula helped to prolong the retention of ANA *in vivo*. Our results went in hand with previous reports that the C_max_ and V_z/F_ of ANA were significantly attenuated and the clearance rate (CL) was decreased responsible for prolonged MRT in the combination of core drug pair and other herbs in JGL formula (Zheng et al., 2022). These results suggested that the maintenance of ANA, which characterized in terms of duality of efficacy and toxicity, at an appropriated lower level with a longer retention time in plasma might contribute to its expected therapeutic actions with fewer side effects. Notably, the rats treated with combination of AC and CH in our study demonstrated significant reduction in the AUC of ANA and this result was inconsistent with previous study that the core drug pair combined with other herbs in JGL showed increased tendency in the AUC of ANA (Zheng et al., 2022). Some possible reasons for this discrepancy are (1) taking different herbal medicines as objects, that *A. chinense* for the present study and the core drug pair for the previous study, in which the active components of *S. cuneate* may decrease the absorption and slow down the metabolism of the active components (ANA) of *A. chinense* in rats, (2) different dosage of *A. chinense,* that the dose used in the previous study is lower than the toxic dose in the present study. In addition to anabasine, we also compared the pharmacokinetic properties of venoterpine (VEN), another main component of *A. chinense*, in rats administrated with AC and combination of AC and CH. The continuous treatment of herbs for 14 days led to marked reductions in the C_max_, AUC_0-t_ and AUC_0-∞_ of VEN following the co-administration of AC and CH in comparison with AC alone. These results indicated that the absorption and exposure level of VEN in rats was decreased through the interaction between different medicinal materials in JGL formula, thus reducing the frequency of *A. chinense* induced toxicity.

Pharmacokinetic herb-herb interactions can be partly explained by the regulation of cytochrome P450 enzymes. Cytochrome P450 enzymes are a diverse group of catalytic enzymes in the liver and can be involved in the biotransformation of approximately 75% of clinical drugs (Rittle and Green, 2010). The enzyme activity as well as the induction and inhibition of these enzymes are main parameters for drug metabolism and have been widely implicated in the toxification and detoxification processes of a variety of chemical agents (Guengerich, 2008). In theory, human *Cyp1a2, Cyp2d6, Cyp2e1*, and *Cyp3a4* correspond to rat *Cyp1a2, Cyp2d4, Cyp2e1*, and *Cyp3a1* (Ma et al., 2019). Therefore, we detected the mRNA expression levels of *Cyp1a2, Cyp2d4, Cyp2e1*, and *Cyp3a1* in the livers of rats from different treatments to explain the regulatory effect of CH on the pharmacokinetics process of AC. AC treatment significantly downregulated the expression of these CYP enzymes when compared to those of the controls. The AC-induced reduction in the expression levels of *Cyp1a2*, *Cyp2d4*, *Cyp2e1*, and *Cyp3a1* mRNA was markedly ameliorated when rats were administered with both AC and the compatible herbs of JGL. Although the specific activity of CYPs was not detected, our present results suggested that these four CYP isoforms might be associated with the mechanisms generated by combining *A. chinense* and the compatible herbs of JGL and that these effects were generated by regulatory effects in the pharmacokinetics process.

In addition to cytochrome P450 enzymes, the intestinal metabolism also plays an essential role in the absorption of orally administered drugs through the intestinal microbiota (López-Yerena et al., 2020). This dynamic intestinal absorption process is regarded as an important factor that explains the compatibility of TCM and ethnomedicines (Wang et al., 2022). A previous study demonstrated that some toxic alkaloids from TCM underwent hydroxylation, deoxygenation, demethylation, deoxidation and demethylation and ester hydrolysis through the intestinal bacteria, thus reducing their toxicity. Nicotin, an alkaloid with a similar chemical structure to anabasine and venoterpine is predominantly transported across Caco-2 cell monolayers in a unidirectional mode by pH-dependent specific transport systems in a manner that corresponds to its intestinal secretion (Fukada et al., 2002). The anti-rheumatic arthritis fraction (ARF) of *G. leucocarpa* exhibited good membrane permeability and strong intestinal absorption characteristics by virtue of active transport and passive diffusion. In addition, three main active ingredients of ARF were demonstrated to be the substrates of P-glycoprotein and may further affect the intestinal absorption characteristics of medicines and herbs (Wang et al., 2022). These previous studies suggested that the complex composition of TCM and the interaction between multiple constituents could result in different absorption levels of active components in the intestinal segments. In the present study, the single and continuous administration of AC and CH led to a reduction in the *in vivo* exposure levels of ANA and VER when compared to single AC treatment. This led us to hypothesize that the components of CH might regulate the intestinal metabolism of anabasine and venoterpine to influence their absorption characteristics. Further metabolic experiments involving anabasine and venoterpine are needed to test this hypothesis.

Collectively, the data described herein are valuable and improve our understanding of the mechanisms by which the compatible herbs of JGL can inhibit the neurotoxicity induced by *A. chinense* to some extent. However, there are some issues that need to be investigated in future studies. Firstly, in addition to the changes in the mRNA expression levels of CYPs, we need to investigate how the compatible herbs of JGL can specifically modulate the activity of CYPs and intestinal metabolism, and then result in detoxication. Secondly, we need to identify the exact ingredients of the compatible herbs of JGL that interact with the active components of *A. chinense*, such as anabasine or other chemicals. According to the present chemical analysis, there were many different types of compounds in JGL, including alkaloids, organic acids, phenolics and saponins, which may have synergistic interactions among them. Previous works have reported that the toxicity of alkaloids in herbal medicines can be attenuated after being combined with other herbs containing saponins or organic acids. For example, the detoxification mechanisms of ginseng to aconitine was that ginsenoside Rg1 could regulate the ion channels pathway (Xu et al., 2022), accelerate the metabolism of aconitine and promote the absorption of benzoylaconine (Xu et al., 2020). Glycyrrhizic acid and liquiritigenin from *Glycyrrhiza uralensis* alleviated *Semen Strychni*-induced acute neurotoxicity through regulating high mobility group protein B1 (HMGB1) related pathway (Duan et al., 2022). Further research needs to investigate the interaction between alkaloids of AC and other chemical constituents from CH, which may attenuate the toxicity of *A. chinense* by modifying solubility, absorption or metabolism of its active alkaloids. Thirdly, transcriptomics and proteomics assays are required to unravel the possible involvement of other mediators underlying the neurotoxic action of AC when combined with other compatible herbs.

## 5 Conclusion

Our data demonstrate that the compatible herbs of JGL formula lead to a reduction in neurotoxicity induced by *A. chinense*. Furthermore, this combination inhibited oxidative damage, reduced the levels of neurotoxic biomarkers, regulated the abnormal levels and metabolisms of neurotransmitters, as well as modulated the pharmacokinetic processes of the active components of *A. chinense* by changing the levels of exposure to chemicals and by ameliorating the downregulation of the expression of cytochrome P450. This investigation provided experimental data for clinical application of JGL formula and its herbal compatibility.

## Data Availability

The original contributions presented in the study are included in the article/Supplementary Material, further inquiries can be directed to the corresponding author.
