# Supplementary material for: Protective effect of compatible herbs in Jin-Gu-Lian formula against Alangium chinense-induced neurotoxicity via oxidative stress, neurotransmitter metabolisms, and pharmacokinetics
Source: Front Pharmacol. 2023 Feb 16;14:1133982. doi: 10.3389/fphar.2023.1133982 (PMC9977795; doi:10.3389/fphar.2023.1133982)
Supplement: Supplementary file 1 [file DataSheet1.DOCX]

**Supplementary Materials**

1. **Supplementary Methods:**
   1. The primer pairs used for analysis of the mRNA levels of cytochrome P450 in the liver

**Table S1. Primer sequences used in this study**

| **Human genes** | **Primer sequence** | **Length (bp)** |
| --- | --- | --- |
| cytochrome P450, family 1, subfamily a, polypeptide 2 (Cyp1a2) | Forward: 5′-CATAGCCTCAGACCCCACAT-3′ | 165 |
|  | Reverse: 5′-ATGGCTCCGATGACATTAGC-3′ |  |
| cytochrome P450, family 3, subfamily a, polypeptide 1 (Cyp3a1) | Forward: 5′-CGTTCACCAGTGGAAGACTCA-3′ | 112 |
|  | Reverse: 5′- ACTTCTTTCACAGGGACAGGT-3′ |  |
| cytochrome P450, family 2, subfamily d, polypeptide 4 (Cyp2d4) | Forward: 5′- GTGCTGCCTTCGCTGACCATAG-3′ | 173 |
|  | Reverse: 5′- TCCAGATTCCTCCTCAAGAGT-3′ |  |
| cytochrome P450, family 2, subfamily e, polypeptide 1 (Cyp2e1) | Forward: 5′- CAGGAAAGCGTGTGTGTGTT-3′ | 146 |
|  | Reverse: 5′- ACTGCCAAAGCCAACTGTG-3′ |  |
| Actin, beta (Actb) | Forward: 5′- CCAGATCATGTTTGAGACCTTCAA-3′ | 87 |
|  | Reverse: 5′- GTGGTACGACCAGAGGCATACA-3′ |  |

- 1. Pharmacokinetic test
     1. Working solution and sample solution

Appropriate amounts of ANA and VEN standards were used to accurately weigh and prepare stock solutions with a 1 mg/mL concentration in methanol. The ANA and VEN stock solutions were mixed and diluted with methanol to obtain a series of mixed standard curve working solutions with ANA and VEN concentrations both of 5~1000 ng/mL. The lower limit of quantitation (LLOQ) and low, medium, high quality control (QC) working solutions with ANA and VEN concentrations both of 5, 10, 100, and 750 ng/mL. An internal standard (IS) stock solution with a 1 mg/mL concentration with methanol was prepared with an appropriate amount of NIC. The IS stock solution was then diluted with dichloromethane into an IS working solution with a 10 ng/mL final NIC concentration. All working solutions were stored at −80°C.

Five microliters of the standard working solution, LLOQ and low, medium, and high QC working solutions were mixed with 45 μL of blank biological sample to prepare standard curve, LLOQ and QC samples.

- - 1. Methodology validation

The developed LC-MS/MS method was validated per the Food and Drug Administration guidelines for industrial bioanalytical method validation procedures. Including specificity, LLOQ, linearity, inter- and intra-day precision and accuracy, dilution reliability, matrix effect, extraction recovery, and stability of ANA and VEN in plasma.

1. **Supplementary Results: Establishment of the determination of ANA and VEN and IS in plasma samples**

As shown in Figure S1, under the analysis conditions, the retention times of ANA, VEN, and IS in the biological samples were 1.46, 1.46, and 1.45 min, respectively. There were no endogenous peaks in the analytes and IS retention times among the blank plasma. This method has good specificity and selectivity. After being determined by this analytical method, ANA and VEN in plasma sample both showed a good linear relationship with linear range of 0.5~100 ng/mL using 1/X^2^ weighting. The lower limit of quantification (LLOQ) for ANA and VEN was 0.5 ng/mL. ANA’s intra- and inter-day precision (RSD) and accuracy (RE) in plasma samples were ranged from 1.18% to 8.52% and 94.30% to 105.26%, respectively. VEN’s intra- and inter-day precision and accuracy in plasma samples were ranged from 1.61% to 8.39% and 88.50% to 93.30%, respectively. The mean extraction recovery of all analytes was from 40.25% to 93.97%, and the RSD of relative matrix effects was within 1.60%~3.70%. The RSD of dilution procedure was no more than 2.52% and accuracy was 95.21~105.87%. The RSD range of ANA and VEN was from 1.67% to 9.20%, the deviation values was ranged from -5.86% to 7.17%。These indicated that ANA and VEN in rat plasma samples were stable at room temperature (25 °C) for 2 h, in the autosampler at 6 °C for 24 h, after two freeze (–80 °C)-thaw(room temperature) cycles, and in long-term storage at –80 °C for 30 days. The detailed method validation results were shown in Supplementary Tables S2~S8.


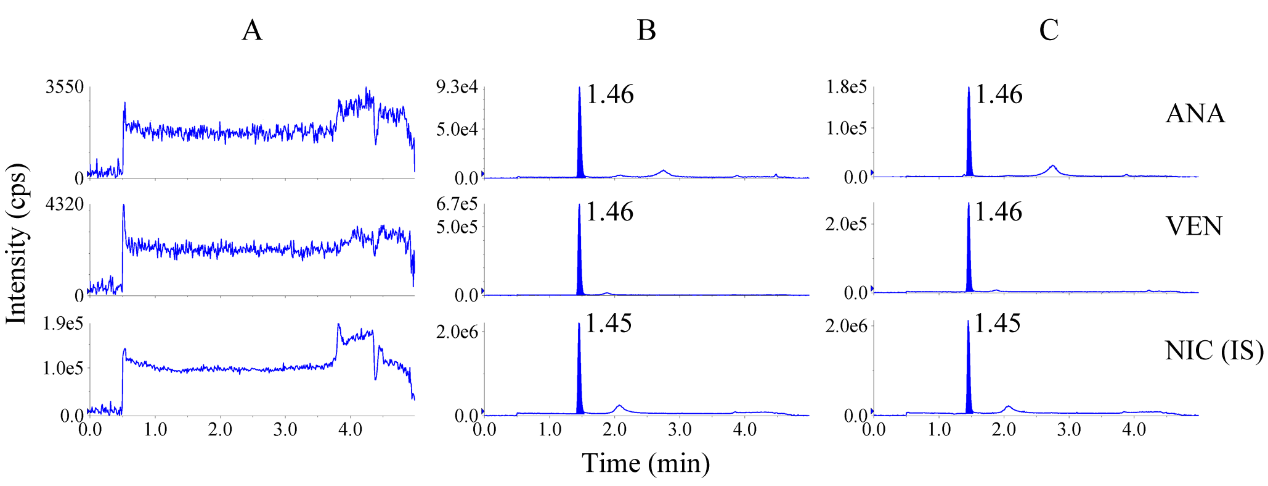


**Figure S1. Chromatograms for anabasine (ANA), venoterpine (VEN) and nicotine (internal standard, IS).** A, blank plasma; B, plasma spiked with ANA (10 ng/mL), VEN (10 ng/mL) and IS; C, plasma samples 5 min after 14 days of the continuous administration of AC and CH (30 g + 30 g crude drugs/kg).

Table S2. Linear equations, the linear range for LC-MS/MS analysis of ANA, VEN in rat plasma.

| Analytes | Calibration curves | Linear range  (ng/mL) | Correlation  coefficients (r) |
| --- | --- | --- | --- |
| ANA | y=0.00340x+0.0121 | 0.5~100 | 0.9992 |
| VEN | y=0.0298x+0.00290 | 0.5~100 | 0.9950 |

Table S3. Precision and accuracy of ANA and VEN in rat plasma.

| Analytes | Nominal  concentration  (ng/mL) | Intra-day (n=6) | | | Inter-day (n=6×3) | | | |  |
| --- | --- | --- | --- | --- | --- | --- | --- | --- | --- |
|  |  | Measured  concentration ^*^  (ng/mL) | Precision  (RSD%) | Accuracy  (%) | | Measured  concentration ^*^  (ng/mL) | Precision  (RSD%) | Accuracy  (%) | |
| ANA | 0.5 | 0.50±0.02 | 4.54 | 100.40 | | 0.53±0.03 | 6.32 | 105.26 | |
|  | 1.0 | 0.94±0.04 | 4.69 | 94.30 | | 0.99±0.07 | 7.09 | 99.11 | |
|  | 10.0 | 10.35±0.88 | 8.52 | 103.51 | | 10.21±0.84 | 8.19 | 102.14 | |
|  | 75.0 | 77.60±0.91 | 1.18 | 103.46 | | 75.60±2.40 | 3.18 | 100.80 | |
| VEN | 0.5 | 0.50±0.04 | 8.39 | 100.77 | | 0.50±0.04 | 8.28 | 99.16 | |
|  | 1.0 | 0.97±0.02 | 2.09 | 96.92 | | 0.99±0.03 | 2.89 | 98.82 | |
|  | 10.0 | 10.25±0.66 | 6.43 | 102.54 | | 10.68±0.64 | 6.01 | 106.83 | |
|  | 75.0 | 66.38±1.07 | 1.61 | 88.50 | | 69.98±2.86 | 4.09 | 93.30 | |

^*^ Mean ± standard deviation. RSD: relative standard deviation

Table S4. Matrix effect and extraction recovery of ANA and VEN in rat plasma (n=6).

| Analytes | Nominal  concentration  (ng/mL) | Matrix effect | | Extraction recovery | |
| --- | --- | --- | --- | --- | --- |
|  |  | Mean±SD (%) | RSD% | Mean±SD (%) | RSD% |
| ANA | 1.0 | 181.78±6.72 | 3.70 | 93.97±5.66 | 6.03 |
|  | 75.0 | 97.94±1.56 | 1.60 | 43.17±3.30 | 7.65 |
| VEN | 1.0 | 93.45±2.23 | 2.39 | 40.25±1.44 | 3.58 |
|  | 75.0 | 96.93±2.25 | 2.32 | 42.82±3.55 | 8.29 |

Table S5. Dilution reliability of ANA and VEN in rat plasma (Mean ± SD, n=6).

| Analytes | Dilution  factor | Concentration (ng/mL) | | Precision  (RSD, %) | Accuracy  (%) |
| --- | --- | --- | --- | --- | --- |
|  |  | Nominal | Measured |  |  |
| ANA | 5 | 375.00 | 397.02 ± 10.01 | 2.52 | 105.87 |
| VEN | 5 | 375.00 | 357.04 ± 8.88 | 2.49 | 95.21 |

Table S6. Stability of ANA and VEN in rat plasma (n=6).

| Analytes | Nominal  concentration  (ng/mL) | Autosampler (6 °C) 24 h | | | Room temperature (25 °C) 2 h | | | Freeze-thaw 2 times | | | Long-term (−80℃, 30 days) | | |
| --- | --- | --- | --- | --- | --- | --- | --- | --- | --- | --- | --- | --- | --- |
|  |  | Measured  concentration ^*^  (ng/mL) | RSD% | Accuracy  (%) | Measured  concentration ^*^  (ng/mL) | Measured  concentration ^*^  (ng/mL) | RSD% | Accuracy  (%) | Measured  concentration ^*^  (ng/mL) | Measured  concentration ^*^  (ng/mL) | RSD% | Accuracy  (%) | Measured  concentration ^*^  (ng/mL) |
| ANA | 1.0 | 0.99±0.09 | 9.01 | 99.05 | 1.02±0.05 | 0.99±0.09 | 9.01 | 99.05 | 1.02±0.05 | 0.99±0.09 | 9.01 | 99.05 | 1.02±0.05 |
|  | 10.0 | 10.27±0.94 | 9.20 | 102.67 | 10.33±0.77 | 10.27±0.94 | 9.20 | 102.67 | 10.33±0.77 | 10.27±0.94 | 9.20 | 102.67 | 10.33±0.77 |
|  | 75.0 | 77.12±2.32 | 3.01 | 102.83 | 78.64±1.31 | 77.12±2.32 | 3.01 | 102.83 | 78.64±1.31 | 77.12±2.32 | 3.01 | 102.83 | 78.64±1.31 |
| VEN | 1.0 | 1.02±0.03 | 2.79 | 102.20 | 1.04±0.02 | 1.02±0.03 | 2.79 | 102.20 | 1.04±0.02 | 1.02±0.03 | 2.79 | 102.20 | 1.04±0.02 |
|  | 10.0 | 10.95±0.41 | 3.74 | 109.48 | 10.99±0.55 | 10.95±0.41 | 3.74 | 109.48 | 10.99±0.55 | 10.95±0.41 | 3.74 | 109.48 | 10.99±0.55 |
|  | 75.0 | 71.37±2.58 | 3.61 | 95.15 | 72.24±1.68 | 71.37±2.58 | 3.61 | 95.15 | 72.24±1.68 | 71.37±2.58 | 3.61 | 95.15 | 72.24±1.68 |

^*^ Mean ± standard deviation. RSD: relative standard deviation

Table S7. Plasma concentration of ANA and VEN at different times after single administration of AC (30 g crude drugs/kg), AC and CH (30 g + 30 g crude drugs/kg) in rats.

|  | Analytes | Time  (h) | Concentration (ng/mL) | | | | | |
| --- | --- | --- | --- | --- | --- | --- | --- | --- |
|  |  |  | 1 | 2 | 3 | 4 | 5 | Mean±SD |
| Single administration of AC | ANA | 0 | ND | ND | ND | ND | ND | —— |
|  |  | 0.083 | 36.99 | 12.56 | 38.25 | 7.85 | 10.36 | 21.20±15.09 |
|  |  | 0.167 | 64.60 | 34.25 | 53.00 | 38.27 | 66.22 | 51.27±14.69 |
|  |  | 0.333 | 106.06 | 74.51 | 93.20 | 85.67 | 123.31 | 96.55±18.85 |
|  |  | 0.667 | 138.23 | 134.16 | 120.53 | 115.92 | 129.88 | 127.74±9.32 |
|  |  | 1 | 176.60 | 136.63 | 67.95* | 140.58 | 177.08 | 157.72±22.14 |
|  |  | 2 | 169.62 | 131.09 | 167.23 | 133.43 | 141.12 | 148.50±18.59 |
|  |  | 4 | 95.77 | 83.29 | 79.60 | 106.52 | 49.29 | 82.89±21.59 |
|  |  | 6 | 36.06 | 59.28 | 45.92 | 66.86 | 30.10 | 47.64±15.41 |
|  |  | 8 | 32.05 | 28.07 | 21.58 | 37.89 | 17.97 | 27.51±7.98 |
|  |  | 10 | 24.51 | 20.10 | 14.87 | 23.03 | 18.62 | 20.22±3.79 |
|  |  | 12 | 15.78 | 14.06 | 10.13 | 15.38 | 9.45 | 12.96±2.97 |
|  |  | 24 | 4.26 | 1.58 | ND | 0.38 | ND | 2.07±1.99 |
|  | VEN | 0 | ND | ND | ND | ND | ND | —— |
|  |  | 0.083 | 3.31 | 1.27 | 3.09 | 1.71 | 1.76 | 2.23±0.91 |
|  |  | 0.167 | 5.08 | 2.33 | 2.58 | 3.08 | 3.68 | 3.35±1.10 |
|  |  | 0.333 | 6.69 | 3.55 | 4.53 | 5.10 | 4.48 | 4.87±1.16 |
|  |  | 0.667 | 7.32 | 2.76 | 4.77 | 5.81 | 3.74 | 4.88±1.78 |
|  |  | 1 | 8.09 | 2.40 | 4.50 | 6.42 | 3.51 | 4.98±2.28 |
|  |  | 2 | 6.46 | 1.18 | 3.57 | 4.53 | 1.51 | 3.45±2.19 |
|  |  | 4 | 1.71 | 0.46 | 0.77 | 2.65 | 0.45 | 1.21±0.96 |
|  |  | 6 | 0.81 | 0.08 | 0.26 | 0.45 | 0.09 | 0.34±0.30 |
|  |  | 8 | 0.25 | 0.08 | 0.09 | 0.21 | 0.05 | 0.14±0.09 |
|  |  | 10 | 0.10 | 0.05 | 0.05 | 0.04 | 0.04 | 0.05±0.02 |
|  |  | 12 | 0.04 | 0.03 | 0.01 | 0.05 | 0.03 | 0.03±0.01 |
|  |  | 24 | 0.02 | 0.01 | 0.01 | 0.02 | 0.01 | 0.01±0.01 |
| Single administration of AC and CH | ANA | 0 | ND | ND | ND | ND | ND | —— |
|  |  | 0.083 | 0.55 | 0.10 | 5.14 | 3.41 | 8.07 | 3.45±3.31 |
|  |  | 0.167 | 8.67 | 19.57 | 26.12 | 30.82 | 33.37 | 23.71±9.91 |
|  |  | 0.333 | 35.19 | 22.32 | 42.71 | 47.56 | 45.74 | 38.70±10.30 |
|  |  | 0.667 | 67.25 | 75.83 | 87.34 | 83.76 | 82.42 | 79.32±7.93 |
|  |  | 1 | 80.90 | 115.84 | 56.18 | 129.00 | 98.85 | 96.15±28.74 |
|  |  | 2 | 111.52 | 141.48 | 25.97 | 118.18 | 109.62 | 101.35±44.01 |
|  |  | 4 | 92.45 | 89.62 | 76.16 | 94.22 | 73.75 | 85.24±9.57 |
|  |  | 6 | 60.97 | 39.30 | 43.37 | 53.57 | 81.79 | 55.80±16.84 |
|  |  | 8 | 42.89 | 28.13 | 44.75 | ND | 23.42 | 34.80±10.62 |
|  |  | 10 | 23.95 | 16.25 | 8.76 | 21.78 | 14.99 | 17.14±5.99 |
|  |  | 12 | 23.44 | 12.82 | 7.86 | 3.58 | 5.64 | 10.67±7.92 |
|  |  | 24 | 5.27 | 5.11 | 3.27 | 5.66 | ND | 4.83±1.06 |
|  | VEN | 0 | ND | ND | ND | ND | ND | —— |
|  |  | 0.083 | 0.25 | 0.29 | 0.62 | 0.83 | 0.69 | 0.54±0.26 |
|  |  | 0.167 | 0.61 | 1.09 | 1.23 | 1.78 | 1.57 | 1.25±0.45 |
|  |  | 0.333 | 1.17 | 2.14 | 2.22 | 2.33 | 2.03 | 1.98±0.46 |
|  |  | 0.667 | 1.12* | 1.92 | 2.18 | 2.51 | 1.79 | 2.10±0.32 |
|  |  | 1 | 1.17 | 2.26 | 2.31 | 3.32 | 2.07 | 2.23±0.77 |
|  |  | 2 | 0.79 | 1.11 | 1.18 | 1.73 | 2.16 | 1.39±0.55 |
|  |  | 4 | 0.31 | 0.33 | 0.44 | 0.97 | 1.07 | 0.62±0.37 |
|  |  | 6 | 0.10 | 0.06 | 0.17 | 0.18 | 0.47 | 0.20±0.16 |
|  |  | 8 | 0.06 | 0.06 | 0.15 | ND | 0.15 | 0.10±0.05 |
|  |  | 10 | 0.06 | 0.02 | 0.10 | 0.07 | 0.02 | 0.05±0.03 |
|  |  | 12 | 0.04 | 0.03 | 0.02 | 0.05 | 0.02 | 0.03±0.01 |
|  |  | 24 | 0.02 | 0.03 | 0.01 | 0.09* | 0.01 | 0.02±0.01 |

ND: Failed to check out. *: Abnormal value, not calculated.

Table S8. Plasma concentration of ANA and VEN at different times after 14 days of continuous administration of AC (30 g crude drugs/kg), AC and CH (30 g + 30 g crude drugs/kg) in rats.

|  | Analytes | Time  (h) | Concentration (ng/mL) | | | | | |
| --- | --- | --- | --- | --- | --- | --- | --- | --- |
|  |  |  | 1 | 2 | 3 | 4 | 5 | Mean±SD |
| 14 days of continuous administration of AC | ANA | 0 | 24.88 | 11.32 | 11.38 | 10.72 | 3.57 | 12.37±7.73 |
|  |  | 0.083 | 27.21 | 21.69 | 28.17 | 21.74 | 1.06 | 19.97±10.99 |
|  |  | 0.167 | 111.41 | 71.63 | 58.10 | 62.48 | 79.77 | 76.68±21.14 |
|  |  | 0.333 | 140.18 | 70.89 | 96.11 | 91.63 | 151.06 | 109.97±34.12 |
|  |  | 0.667 | 169.55 | 126.99 | 123.85 | 114.83 | 146.89 | 136.42±21.91 |
|  |  | 1 | 191.73 | 178.86 | 148.27 | 153.40 | 192.46 | 172.94±20.97 |
|  |  | 2 | 252.07 | 204.70 | 196.89 | 177.42 | 191.99 | 204.62±28.33 |
|  |  | 4 | 108.82 | 141.91 | 101.14 | 108.71 | 84.99 | 109.12±20.75 |
|  |  | 6 | 37.67 | 37.26 | 43.85 | 53.01 | 54.75 | 45.31±8.27 |
|  |  | 8 | 26.61 | 19.70 | 35.80 | 34.18 | 29.72 | 29.20±6.43 |
|  |  | 10 | 12.14 | 22.36 | 23.82 | 26.30 | 20.24 | 20.97±5.41 |
|  |  | 12 | 15.04 | 8.22 | 19.20 | 13.03 | 17.78 | 14.65±4.32 |
|  |  | 24 | 0.72 | 3.18 | 0.92 | 2.48 | 0.95 | 1.65±1.11 |
|  | VEN | 0 | ND | ND | ND | ND | ND | —— |
|  |  | 0.083 | 3.73 | 1.18 | 2.50 | 2.28 | 0.43 | 2.02±1.27 |
|  |  | 0.167 | 7.23 | 3.45 | 4.15 | 4.89 | 6.28 | 5.20±1.54 |
|  |  | 0.333 | 9.14 | 3.48 | 5.89 | 6.54 | 7.80 | 6.57±2.13 |
|  |  | 0.667 | 9.64 | 5.19 | 7.55 | 7.90 | 8.96 | 7.85±1.70 |
|  |  | 1 | 10.68 | 6.12 | 9.50 | 10.13 | 6.63 | 8.61±2.09 |
|  |  | 2 | 10.05 | 5.46 | 8.75 | 9.32 | 3.85 | 7.49±2.69 |
|  |  | 4 | 3.92 | 1.24 | 3.46 | 4.35 | 1.10 | 2.81±1.53 |
|  |  | 6 | 1.12 | 0.45 | 1.42 | 1.50 | 0.28 | 0.96±0.56 |
|  |  | 8 | 0.34 | 0.10 | 0.46 | 0.56 | 0.03 | 0.30±0.23 |
|  |  | 10 | 0.32 | ND | 0.17 | 0.22 | ND | 0.23±0.08 |
|  |  | 12 | ND | ND | 0.03 | 0.09 | ND | 0.06±0.04 |
|  |  | 24 | ND | ND | ND | ND | ND | —— |
| 14 days of continuous of AC and CH | ANA | 0 | 8.49 | 10.00 | 8.49 | 9.75 | 5.98 | 8.54±1.59 |
|  |  | 0.083 | 16.74 | 14.15 | 19.04 | 16.46 | 25.67 | 18.41±4.41 |
|  |  | 0.167 | 41.19 | 48.32 | 60.58 | 44.92 | 43.19 | 47.64±7.69 |
|  |  | 0.333 | 63.69 | 83.88 | 59.67 | 68.63 | 46.25 | 64.42±13.69 |
|  |  | 0.667 | 103.06 | 127.81 | 99.39 | 93.10 | 92.82 | 103.24±14.41 |
|  |  | 1 | 75.66* | 61.17* | 75.45* | 115.42 | 113.63 | 114.53±1.26 |
|  |  | 2 | 123.65 | 148.07 | 126.92 | 116.61 | 122.28 | 127.51±12.09 |
|  |  | 4 | 72.36 | 70.05 | 70.07 | 75.58 | 65.94 | 70.80±3.54 |
|  |  | 6 | 39.62 | 32.75 | 38.79 | 39.28 | 48.30 | 39.75±5.55 |
|  |  | 8 | 6.50* | 3.09* | 6.89* | 22.28 | 30.32 | 26.30±5.68 |
|  |  | 10 | 2.62* | 3.65* | 3.66* | 0.25* | 18.38 | 18.38±0.00 |
|  |  | 12 | 14.57 | 12.50 | 13.87 | 10.92 | 20.28 | 14.43±3.56 |
|  |  | 24 | 0.42 | 0.28 | 0.29 | 0.01 | 1.06 | 0.41±0.39 |
|  | VEN | 0 | ND | ND | ND | ND | ND | —— |
|  |  | 0.083 | 1.40 | 1.08 | 1.44 | 1.12 | 1.84 | 1.38±0.31 |
|  |  | 0.167 | 2.36 | 2.47 | 3.49 | 2.33 | 2.63 | 2.66±0.48 |
|  |  | 0.333 | 4.42 | 4.38 | 3.54 | 3.55 | 4.55 | 4.09±0.50 |
|  |  | 0.667 | 5.33 | 5.44 | 5.28 | 5.38 | 5.04 | 5.29±0.15 |
|  |  | 1 | 5.98 | 5.44 | 6.11 | 4.84 | 5.46 | 5.56±0.51 |
|  |  | 2 | 3.93 | 3.31 | 4.04 | 3.80 | 4.83 | 3.98±0.55 |
|  |  | 4 | 1.27 | 0.40 | 1.26 | 1.38 | 1.82 | 1.23±0.52 |
|  |  | 6 | 0.37 | ND | 0.38 | 0.28 | 0.89 | 0.48±0.28 |
|  |  | 8 | ND | ND | ND | 0.04 | 0.24 | 0.14±0.14 |
|  |  | 10 | 0.04 | ND | ND | ND | 0.05 | 0.04±0.01 |
|  |  | 12 | ND | ND | ND | ND | ND | —— |
|  |  | 24 | ND | ND | ND | ND | ND | —— |

ND: Failed to check out. *: Abnormal value, not calculated.
